# The Build-Up of Population Genetic Divergence along the Speciation Continuum during a Recent Adaptive Radiation of *Rhagoletis* Flies

**DOI:** 10.3390/genes13020275

**Published:** 2022-01-30

**Authors:** Thomas H. Q. Powell, Glen Ray Hood, Meredith M. Doellman, Pheobe M. Deneen, James J. Smith, Stewart H. Berlocher, Jeffrey L. Feder

**Affiliations:** 1 Department of Biological Sciences, Binghamton University, State University of New York, Binghamton, NY 13902, USA; pdeneen1@binghamton.edu; 2 Department of Biological Sciences, Wayne State University, Detroit, MI 48202, USA; glenrayhood@wayne.edu; 3 Department of Biological Sciences, University of Notre Dame, Notre Dame, IN 46556, USA; mdoellma@nd.edu (M.M.D.); Jeffrey.L.Feder.2@nd.edu (J.L.F.); 4 Department of Entomology & Lyman Briggs College, Michigan State University, East Lansing, MI 48824, USA; jimsmith@msu.edu; 5 Department of Entomology, University of Illinois Urbana-Champaign, Urbana, IL 61801, USA; stewartb@illinois.edu; 6 Environmental Change Initiative, & Advanced Diagnostics and Therapeutics, University of Notre Dame, Notre Dame, IN 46556, USA

**Keywords:** ecological speciation, clines, gene flow, hybridization, sympatric speciation, Tephritidae

## Abstract

New species form through the evolution of genetic barriers to gene flow between previously interbreeding populations. The understanding of how speciation proceeds is hampered by our inability to follow cases of incipient speciation through time. Comparative approaches examining different diverging taxa may offer limited inferences, unless they fulfill criteria that make the comparisons relevant. Here, we test for those criteria in a recent adaptive radiation of the *Rhagoletis pomonella* species group (RPSG) hypothesized to have diverged in sympatry via adaptation to different host fruits. We use a large-scale population genetic survey of 1568 flies across 33 populations to: (1) detect on-going hybridization, (2) determine whether the RPSG is derived from the same proximate ancestor, and (3) examine patterns of clustering and differentiation among sympatric populations. We find that divergence of each in-group RPSG taxon is occurring under current gene flow, that the derived members are nested within the large pool of genetic variation present in hawthorn-infesting populations of *R. pomonella*, and that sympatric population pairs differ markedly in their degree of genotypic clustering and differentiation across loci. We conclude that the RPSG provides a particularly robust opportunity to make direct comparisons to test hypotheses about how ecological speciation proceeds despite on-going gene flow.

## 1. Introduction

Speciation is the process responsible for generating new twigs on the tree of life. New species form as genetically based barriers to gene flow evolve between formerly interbreeding populations [1,2,3]. One broad objective of speciation research is to identify those traits responsible for reproductive isolation (RI) and to discern their evolutionary origins and genetic underpinning [4,5,6]. A second approach to understanding this process is to characterize and quantify patterns of genetic divergence between taxa at different stages of speciation and from these patterns make inferences about the processes driving diversification [7,8,9]. For example, when speciation occurs in the face of documented, on-going gene flow, regions of the genome showing population genetic differentiation above neutral expectations of drift/migration balance may be inferred to contain genes under divergent selection contributing to RI. Analyses of how such patterns change as taxa move further along the speciation continuum can shed light on questions such as the roles that chromosome structure (i.e., chromosomal variants associated with reduced recombination), the genetic architecture of selected phenotypes (i.e., large effect mutations confined to small regions of particular chromosomes versus polygenic quantitative allelic variation distributed throughout the genome), and evolutionary coupling may play in facilitating or constraining speciation. 

However, drawing inferences from patterns of population genetic differentiation is complicated by several factors. One important consideration is the need to confirm that on-going gene flow is actually occurring between taxa. The possibility of incomplete lineage sorting means that shared allelic variation is insufficient to demonstrate that speciation is indeed proceeding in the face of gene flow. Heterogeneous topologies of divergence can still occur between sibling taxa, even in the complete absence of gene flow, driven by differences in post-divergence selection histories or demographics in the diverging taxa [10,11]. Another complication is our inability to track individual speciation events across sufficient time. Much of our understanding of the process of speciation comes from pairwise comparisons between diverging populations or taxa [12,13,14,15,16,17,18]. Most of these systems do not offer a sequential series of comparisons from ecotypes, varieties, or races through to more fully diverged species. Thus, it is difficult to determine whether the traits and processes involved in early stages of divergence may play important roles in catalyzing later stages of speciation. It is similarly difficult to attach importance to a specific pattern of genomic differentiation, as these pairwise comparisons represent static snapshots of dynamic processes. Comparisons across phylogenetically, ecologically, and biogeographically disparate systems are generally unsatisfying. Instead, comparative studies of recent radiations whose members differ in their degree of phenotypic divergence and reproductive isolation may be particularly informative proxies for transitions along the speciation continuum [19,20,21,22,23].

Here, we focus on a set of recently diverged sister taxa, the *Rhagoletis pomonella* species group (RPSG), to test some underlying assumptions of comparative approaches to studying the speciation continuum and examine patterns of population genetic differentiation at different stages of speciation in this system. The most well-studied component of this species group is the apple-infesting host race of *R. pomonella*, which arose as the result of a shift from ancestral hawthorn (*Crataegus* spp.) to introduced apple (*Malus pumila*) in recent historical times, less than 170 years ago [24,25,26]. The RPSG is a model system for ecological speciation [27] comprised of several populations and taxa, ranging from locally adapted host-associated populations through to morphologically and phylogenetically distinct species (Table 1; [28,29,30]). For semantic concision, we will hereafter refer to these host-specific populations simply as ‘taxa,’ without opining on the taxonomic status of these groups. The RPSG taxa are broadly sympatric across much of eastern North America (Figure S1), specialize on non-overlapping sets of host plants, and are reproductively isolated from one another largely by the same set of ecological traits, primarily life history timing and neurophysiological responses to fruit volatiles [31,32,33,34,35,36,37,38,39,40,41]. Moreover, the members of the RPSG may form a nested set of comparisons, as within the clade, each taxon is thought to have been derived from ancestral hawthorn-infesting populations of *R. pomonella* [30]. Previous studies have found that, with the exception of the morphologically distinct *R. cornivora*, mitochondrial and nuclear haplotype variation is shared among members of the RPSG [22,30,37,42,43,44], with differences resulting from frequency of shared variants. This pattern of sequence variation segregating across populations is expected if divergence is occurring under continuous gene flow and renders molecular genetic distance metrics of sequence divergence inadequate to describe the population genetic relationship among taxa. In this study, we use multilocus microsatellite variation, which is particularly well suited to studying cases of recent divergence with on-going gene flow [45,46,47,48] to test whether the RPSG conforms to certain conditions that would make it particularly ideal for studying transitions along the speciation continuum with gene flow and to examine patterns of population genetic differentiation among these taxa. 

The first hypothesis we test is that divergence among members of the RPSG is maintained in the face of on-going gene flow. Establishing the presence of active gene flow is critical for making inferences about the balance of evolutionary forces responsible for a given pattern of differentiation. In systems involving early stages of speciation (i.e., host races or ‘host forms’ *sensu* Funk 2002 [49]), on-going gene flow is often assumed due to a high degree of allelic similarity; if taxa share all of their allelic variation across a number of quickly evolving loci, it is reasonable to infer that any complete cessation of gene flow would have occurred only recently. In the classic apple race story, this hypothesis has been tested directly with mark-recapture studies demonstrating gross migration rates of 4–6% at sympatric sites [50]. In the absence of divergent selection, these migration rates are more than sufficient to homogenize allele frequencies at equilibrium [51]. For taxa further along the speciation continuum, an alternative to these labor-intensive studies (which necessarily become even more labor intensive as migration rates decrease) is to use genotypic information to identify F1 hybrid and backcross individuals in natural populations [22,52]. 

The second hypothesis we address is that the RPSG represents a series of nested taxa that all share hawthorn-infesting *R. pomonella* as their proximate ancestor. With the exception of the morphologically distinct *R. cornivora*, all the taxa in the RPSG in the United States are thought to form a phylogenetic in-group relative to populations of hawthorn-infesting *R. pomonella* in the highlands of Mexico [30,42,43]. Both cDNA and mtDNA sequence data show that none of these in-group taxa appear to be monophyletic with respect to hawthorn-infesting *R. pomonella* in the United States, and haplotypes associated with these taxa are derived from the broad sequence variation within *R. pomonella* [30,44]. Here, we examine this using multilocus microsatellite data from the perspectives of patterns of shared polymorphisms among taxa and population genetic distance networks.

While the proximate divergence of the apple-infesting race almost certainly occurred in a fully sympatric context (the introduced nature of the derived host as well as the historical and current range of the apple race leave little room for doubt), the genetic variation from which apple-adaptive genotypes were composed likely has a more complicated origin [53]. Variation underlying the life history traits of the apple race appears to be drawn from adaptive latitudinal clines present in the hawthorn race, extending from the upper-Midwest to the Gulf Coast, which are a dominant feature of the population genetics of the hawthorn flies [54,55,56]. The co-option of this clinal variation segregating within the hawthorn population was critical for the rapid evolution of the apple race [42]. The source of genetic variation underlying complex ecological traits has important consequences for the likelihood and tempo of ecological divergence [57,58]. The colonization of and adaptation to novel habitats or niches that require multiples axes of ecological divergence and/or polygenic traits is much more likely to occur if adaptive genotypes are drawn primarily from standing genetic variation rather than de novo mutations [59]. The establishment of the latitudinal clines in *R. pomonella* likely predates the radiation of the RPSG [43,60], and thus this ancestral variation may have served as important fodder for natural selection during the emergence of many of these phenologically distinct taxa. Here, we also test for the signature of the co-option of ancestral clinal variation in RPSG taxa.

Lastly, we examine patterns of population genetic differentiation among members of the RPSG that differ in their position along the speciation continuum. We focus on patterns observed between pairs of sympatric populations representing a derived RPSG taxon paired with a local ancestral hawthorn-infesting *R. pomonella* population. Across these sympatric pairs, we compare the patterns of genotypic clustering and the distribution of locus-by-locus differentiation. We also estimate the selection coefficients needed to maintain observed allele frequency differences between population pairs given estimates of gross migration rates. We then use the results of these analyses to discuss the progression of population genetic divergence in the face of gene flow at different stages along the speciation continuum in these flies. 

## 2. Materials and Methods

### 2.1. Sampling of Flies and Microsatellite Genotyping

All insects genotyped in this study were obtained by rearing larvae from infested fruit collected in the field using standard *R. pomonella* collection techniques (see Powell et al. 2014 [37] for detailed description of field collection and laboratory rearing methods). A total of 1568 flies sampled from 33 host-associated populations, including 14 hawthorn-infesting *R. pomonella* populations, six apple-infesting *R. pomonella* populations, four blueberry-infesting *R. mendax* populations, five snowberry-infesting *R. zephyria* populations, one silky dogwood-infesting *R. cornivora* population, and three populations of the unnamed fly infesting flowering dogwood were each scored for 19 microsatellite markers (Table 1; Figure S2). The hawthorn-infesting *R. pomonella* populations include five populations from downy hawthorn, (*C. mollis*, the major native host in the Midwest and Northeast), three from green hawthorn (*C. viridis*, the most widespread host in the South), two each from mayhaw (*C. opaca*), and blueberry hawthorn (*C. brachyacantha*, two alternative hosts in the South), and two from black hawthorn (*C. douglasii*, a major host in the Pacific Northwest). Six populations in total, 2 from apple, 2 from black hawthorn, and 2 from *R. zephyria*, come from the Pacific Northwest, where *R. zephyria* is thought to be native but *R. pomonella* populations were recently introduced in the late 1970s [48,61]. New microsatellite genotype data from *R. mendax*, *R. zephyria*, and *R. cornivora* populations were combined with previously published data for *R. pomonella* [37,56,61,62] and the flowering dogwood fly [22]. 

We PCR amplified and scored 19 microsatellite markers for *R. mendax*, *R. zephyria*, and *R. cornivora* spanning five of the six *R. pomonella* linkage groups [56] (the sixth group is a dot chromosome which currently lacks markers) (Table S1). Complementary primer pairs for the 19 loci, developed by Velez et al. [63], have been used as a core set of markers in several previous studies of the RPSG [22,37,56,60,62,64,65]. This particular set of 19 markers was chosen because they amplify robustly across all of the focal taxa with little evidence of major null alleles in previous studies of members of the RPSG [22,37,48,56,60,61,62,65]. Genomic DNA extraction and PCR amplification methods follow those of Powell et al. [22,37] (see Appendix A). Genotyping was performed on a Beckman-Coulter CEQ8000 (Brea, CA, USA), and microsatellite fragment length polymorphisms were sized using the Fragment Analysis software provided by Beckman-Coulter. A subset of samples from previous studies were run during the genotyping of the new populations to ensure consistency of fragment length estimation, and allele calls were made by re-binning fragment lengths within a database of RPSG microsatellite data (see Appendix A). In the current study, most populations were found to be slightly depauperate of heterozygotes, but with considerable variation across loci (Table S2). For all populations, 95% confidence interval for the inbreeding coefficient *F_IS_* across loci overlapped zero (Table S2), indicating that systematic inbreeding was not likely a major factor shaping the distribution of allelic variation within populations. 

### 2.2. Detection of On-Going Gene Flow

To test for on-going gene flow among members of the RPSG, we used the built-in function of STRUCTURE 2.3.4 [66] for detecting putative parental migrants, hybrids, and backcrosses in cases with strong underlying population structure [22,52,65]. Our approach first involved using STRUCTURE to infer the total number of discrete genotypic clusters within three broad geographic regions (North, South, and Pacific Northwest). We restricted this analysis to these three geographic subsets to help mitigate the confounding factors of strong clinal variation in *R. pomonella* which is a poor fit for the discrete Hardy–Weinberg model of STRUCTURE [22]. The single *R. cornivora* population was included in both the North and South regional analyses because the taxon is found in both regions. For each of these regional analyses, we ran five replicate runs of 500,000 MCMC iterations following a burn-in period of 250,000 iterations using the admixture and correlated allele frequency model of K = 1 to K = the total number of site × host combinations in each region (North = 15, South = 13, PNW = 6). These initial runs were conducted “blind” without a priori population designations for individuals. The best fit K-value was determined by the Δ*K* method of Evanno et al. [67] (Table S3). 

Next, we used the results of these initial blind analyses to provide a priori population designations for STRUCTURE runs to detect migrants and hybrids. For each regional analysis, we conducted three replicate runs under the admixture with correlated allele frequency model at each of three values of the migration prior (MIGPRIOR): 0.01, 0.05 (default), and 0.1. Each run was conducted for 1,000,000 iterations after discarding a burn-in of 500,000 iterations. These options in STRUCTURE produce an output of individuals categorized as belonging to one of four genotypic classes based on posterior probabilities: pure natal-host genotypes, pure parental migrants possessing non-natal host genotypes, F1 hybrids having non-natal host heritage one generation back, or backcrosses having non-natal host heritage two generations back. 

### 2.3. Patterns of Allelic Variation within the RPSG

To confirm that the in-group taxa in the RPSG are indeed a nested set of sibling taxa, each derived from *R. pomonella*, we examined patterns of shared allelic variation among each of the species group members. To quantify and compare levels of polymorphism among members of the RPSG, allelic richness for each population was estimated using a rarefaction approach implemented in ADZE [68] and fitting the rarefaction curves to asymptotic models in R ver. 3.2.2 (R Development Core, Vienna, Austria). Details of the rarefaction methods, curve fitting, and statistical comparison are presented in the Appendix A. We quantified the frequency of private alleles for members of the RPSG relative to putatively ancestral hawthorn-infesting *R. pomonella* (including both northern and southern hawthorn races). We were interested in alleles that were established in one taxon relative to the other and, thus, we did not include singleton mutations found in only one fly or alleles present in only a single geographic site in our estimates of private allele frequencies. Instead, we defined private alleles as fragment-length variants present in at least two populations of a taxon and entirely absent from any of the *R. pomonella* populations surveyed (note that the two-population requirement could not be applied to the single *R. cornivora* population surveyed in this study, and, thus, we used only a single population criterion in this case). 

### 2.4. Ancestral Clinal Variation within the RPSG

In order to understand how the radiation of the RPSG fits into patterns of standing clinal variation within *R. pomonella*, we compared the allele frequencies of the apple race, flowering dogwood fly, *R. mendax*, *R. zephyria*, and *R. cornivora* to the major patterns of latitudinal variation within the hawthorn race (see Appendix A for full details). Briefly, we first collapsed the allelic variation independently at each locus in the putatively ancestral hawthorn race into orthogonal axes by principal components analysis (PCA) and then identified ‘major clinal’ loci for which the first principal component correlated strongly with latitude. We then compared the allelic variation in the derived taxa in the RPSG to the major clinal variation for loci within the hawthorn race via overlap of mean principal component confidence intervals.

### 2.5. Population-Level Clustering within the RPSG

To address the hypothesis that the in-group of the RPSG represent nested sibling taxa and examine broad-scale patterns of differentiation across the speciation continuum, neighbor-joining networks were constructed based on Nei’s D pairwise distance [69] for the 19 microsatellites using the program PowerMarker [70]. Two networks were created for all populations and for only the in-group taxa, with confidence in the modes assessed by consensus among 10,000 bootstrap replicates across loci. These networks are reflective of current population genetic distances among populations, based on allele frequency dissimilarity, that are affected by, but do not necessarily perfectly mirror, past historical relationships. Some population genetic networks can be interpreted from a historical or systematic perspective if they conform to assumptions of ‘treeness,’ typically involving breaks in gene flow with subsequent divergence being driven by clock-like drift and mutation [71]. These assumptions are a poor fit for divergence under protracted or on-going gene flow as is the case with the RPSG. Therefore, these networks cannot be rooted in a historically meaningful way. Instead, these networks describe variation in the dissimilarity of current allele frequencies among populations, which may be driven by a combination of historical contingencies and on-going evolutionary forces.

### 2.6. Patterns of Population Genetic Differentiation at Sympatric Sites

To reduce the confounding effect of geography (latitudinal clines) on patterns of differentiation, we restricted a subset of analyses to independent population pairs co-occurring in sympatry or in close proximity to one another (see Table 1 and Figure S1). Each of the seven pairs consisted of *R. pomonella* and one of the derived RPSG taxa, with each taxon represented once. We used this subset of data to examine patterns of individual-level clustering, the distribution of locus-specific differentiation, and patterns of estimated divergent selection.

We conducted STRUCTURE analyses to determine the strength of genotypic clustering at each of the sympatric comparison sites. Specifically, we ran STRUCTURE for K = 1–2 under the admixture and correlated allele frequencies model. For each value of K, we ran 10 replicates of 750,000 iterations after discarding a burn-in period of 500,000 iterations. The comparison of only two values of K does not allow for Evanno et al. [67] method for determining the best K value. Here, we used the mean Ln likelihood estimates for K = 1 and K = 2 to determine the best fit. 

To examine the locus-by-locus patterns of population genetic differentiation for different members of the RPSG, we calculated Jost’s D_EST_ [72] for the set of paired sympatric populations using the R package ‘diveRsity’ [73]. We used Jost’s D_EST_ because of the high intrapopulation polymorphism previously documented for many of the loci developed for *R. pomonella* [37,48,56], which can be problematic for F_ST_ and other metrics which rely on an additive decomposition of expected heterozygosity [72,74,75]. 

To understand the interaction of evolutionary forces in driving patterns of differentiation in these sympatric population pairs, we estimated the selection coefficients (*s*) needed to maintain the observed allele frequency differences given the estimated gross migration rates between taxa at equilibrium (see Appendix A for migration rates used in this analysis). In order to concentrate on a single defined allele frequency difference at each locus, we simplified the microsatellite data by pooling the alleles that were in higher frequency in each taxon. This resulted in two allele classes for each locus at each site: one class was composed of alleles more common in *R. pomonella* and one of alleles more common in the derived taxon. Previous research on RPSG flies, including the apple race, southern hawthorn races, and the flowering dogwood fly, have found that microsatellite (and allozyme) variation at a locus can be decomposed into major allele classes identified through linkage disequilibrium relationships [22,37,56]. Such classes reflect the same deeply divergent ancestral variation underlying latitudinal clinal patterns and chromosomal inversions [42,43,60]. The analytical model is fully described in the Appendix A. In brief, we used a two-island model of symmetric gross migration with equal population size and assumed a dominance coefficient (*h*) of 0.5 to calculate the selection coefficients needed to produce an exactly compensatory value of Δ*p* compared to homogenizing Δ*p* from migration alone at each locus. This produced two sets of selection coefficients, one describing selection against alleles favored in the ancestral taxon in the derived taxon’s population and one describing selection against alleles favored in the derived taxa in the ancestral population. In some cases, this model produced estimates of *s* greater than 1, indicating that the observed allele frequency difference is too strong to be maintained by selection at equilibrium given the particular model assumptions. We report these extreme *s* values as 1. Based on the considerable assumptions that our model makes, including evolutionary forces acting at equilibrium, examining only direct selection on each locus, the simplicity of symmetric migration rates, and the use of composite allele frequencies for these highly polymorphic loci, we are not suggesting that these estimates represent the true selection regime acting on these populations. Rather, these estimates speak to the general magnitude and distribution of on-going divergent selection required to maintain the observed genetic differentiation within the RPSG in the face of gene flow. 

## 3. Results

### 3.1. Evidence for On-Going Gene Flow 

Within each of the three broad geographic regions (North, South, and Pacific Northwest), blind STRUCTURE analyses supported discrete genotypic clusters corresponding to each of the species-level in-group taxa and *R. cornivora* (Figure S3; Table S3). These genotypic clusters were then used as a priori population designations in the subsequent STRUCTURE analyses. Taxa at the host race level and below were not consistently differentiated across loci to the degree to which the genotypic detection of migrants and hybrids can be well supported. However, evidence from mark–recapture studies places the migration rate between the apple and hawthorn races at sympatric sites at ~4–6% [50]. 

Results from the hybrid detection STRUCTURE analyses were highly consistent across replicates and qualitatively similar for each value of the migration prior. Here, we report the results from the most conservative migration prior value of *m* = 0.01. Previous studies found genotypic evidence for on-going gene flow between *R. pomonella* and the flowering dogwood fly [22] and *R. pomonella* and *R. zephyria* [54,65,76], and here our analyses also detected putative parental migrant, F1, and backcrosses individuals between these taxa and *R. pomonella* (Tables S4–S6; Figure S3). In addition, we also found evidence for on-going bidirectional gene flow between *R. mendax* and *R. pomonella*: one blueberry-hawthorn origin individual in the South had a high posterior probability of being an F1 *R. mendax* hybrid, one apple-origin population fly in the North regional analysis scored as a likely *R. mendax* backcross, and one blueberry-origin fly from Fennville, Michigan scored as an F1 *R. pomonella* hybrid (Tables S4 and S5; Figure S3). We found no evidence of migrants, hybrids, or backcrosses between the morphologically distinct *R. cornivora* and any other taxon. For other pairwise comparisons among taxa other than *R. pomonella*, the only individual with a higher posterior probability of non-natal ancestry was a single *R. zephyria* individual from Polk Co., Minnesota that was categorized as a likely *R. mendax* backcross (Table S4; Figure S3).

### 3.2. Evidence for a Nested Series of Taxa Derived from Hawthorn-Infesting R. pomonella 

#### 3.2.1. Allelic Richness

The majority of allelic variation at the 19 microsatellites scored in this study was shared among the four in-group taxa, *R. pomonella* (both apple- and hawthorn-infesting populations), flowering dogwood flies, *R. mendax*, and *R. zephyria* (see online supplementary allele frequency file). No private alleles were found that differentiated the flowering dogwood fly from *R. pomonella* (Table 2). Private microsatellite alleles were detected, however, distinguishing the other two in-group taxa *R. mendax*, and *R. zephyria* (Table 2). Neither of these taxa possessed fixed differences at any of the 19 loci. *Rhagoletis cornivora* had private alleles at 13 of the 19 loci, including two loci with fixed differences (P3 and P9). *Rhagoletis zephyria* had a greater number of loci showing private alleles than *R. mendax* (nine versus five). The frequency of private alleles was also generally higher for *R. zephyria*, with one locus P7 at 75%. The same locus was also the highest private allele frequency observed for *R. mendax* at 19% (Table 2).

**Figure 1:**
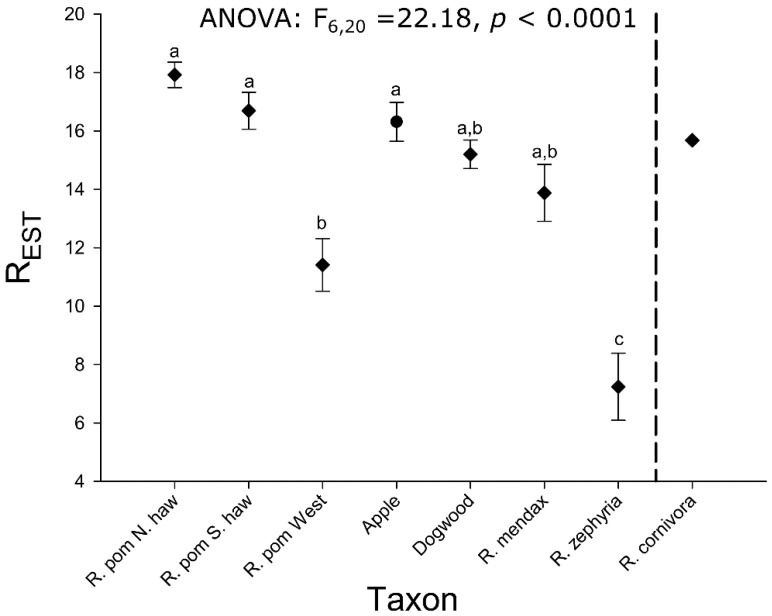
Mean estimated allelic richness (±SE) across 19 microsatellite loci for *R. pomonella* species complex members, based on fitting rarefaction curves to a asymptotic model. Lowercase letters reflect significant differences based on Tukey’s post hoc test. *R. cornivora*, to the right of the dashed lines, lacks error bars and post hoc test significance designations because only a single population of this taxon was analyzed.

Allelic richness varied significantly among taxa in the *R. pomonella* group (Figure 1; ANOVA, F_6,20_ = 22.18; *p* < 0.0001). Except for the recently introduced western populations of flies, *R. pomonella* had the highest estimated allelic richness across loci (Figure 1). This finding is consistent with the hawthorn-infesting *R. pomonella* being ancestral to the rest of the in-group species in the RPSG, although the pattern could also be explained by a higher mutation rate or larger population size in hawthorn flies. The flowering dogwood fly and *R. mendax* each showed slight but non-significant decreases in allelic richness relative to *R. pomonella* (Figure 1). Allelic richness in *R. zephyria* was significantly lower than any other taxon, with a mean value less than half that for native-range *R. pomonella*. The single population of *R. cornivora* tested prevented a statistical comparison. However, allelic richness of the single population surveyed was similar to that of *R. pomonella*, with the mean falling within the confidence intervals of southern hawthorn-infesting *R. pomonella* and the apple race. 

#### 3.2.2. Population-Level Clustering

**Figure 2:**
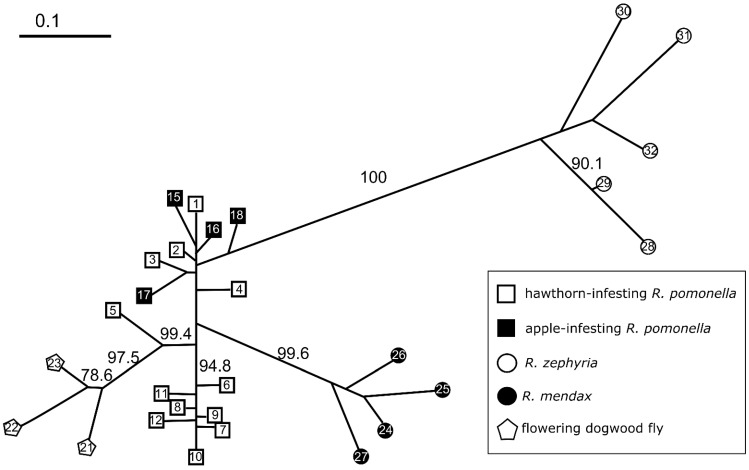
Neighbor-joining network based on Nei’s D (1972) estimated from microsatellite allele frequencies for 19 loci for 32 populations of in-group RPSG taxa. Open squares = hawthorn-infesting *R. pomonella*; closed squares = apple-infesting *R. pomonella*; open circles = snowberry-infesting *R. zephyria*; closed circles = blueberry-infesting *R. mendax*; pentagons = flowering dogwood fly. *R. cornivora* is omitted from this network due to poorly supported positioning and a branch length that obscures the primary features of the in-group network. The full network, including *R. cornivora*, is presented in Figure S4. Bootstrap values come from 10,000 replicates across loci. Numbers within nodes refer to sites listed in Table 1.

The NJ network analyses revealed variation in range-wide population clustering among RPSG taxa (Figure 2). The position of *R. cornivora* was poorly supported in the network (Figure S4), appearing generally equidistant, driven by allele frequencies approaching fixation (Table 2 and online supplementary allele frequency file) from all other host-associated populations. The apparent basal clustering of *R. cornivora* with *R. zephyria* in the network appears to be due to some slight similarity (possibly homoplasic) with *R. zephyria* at only a single locus. At the majority of loci, *R. cornivora* and *R. zephyria* did not share unique alleles relative to *R. pomonella*. The bootstrap support for this association was less than 45%, therefore, we concentrate on patterns observed in networks containing only the in-group taxa (Figure 2). 

The in-group network showed two important features (Figure 2). First, despite the lack of fixed differences, the flowering dogwood fly, *R. mendax*, and *R. zephyria* populations each formed discrete clusters relative to the other *R. pomonella* populations. Beyond this qualitative benchmark, the strength of this pattern increased with the more differentiated taxa. The two named taxa both show higher bootstrap values and considerably longer branch lengths than the flowering dogwood fly (Figure 2). Second, hawthorn-infesting *R. pomonella* populations do not form a discrete cluster relative to the other taxa (Figure 2). Although genetic variation appears to be continuous among northern and southern populations of *R. pomonella* [22,55,62], populations from these regions cluster separately from each other relative to other taxa in the complex, with apple-infesting population interspersed in the northern hawthorn fly cluster. This pattern adds further evidence to the hypothesis that *R. pomonella* was indeed the proximate ancestor to the flowering dogwood fly, *R. zephyria*, and *R. mendax*. 

### 3.3. Evidence for the Role of Clinal Variation in the RPSG

Seven major clinal loci were detected in hawthorn-infesting populations of *R. pomonella* based on PCA analysis: P71, P37, P70, P80, P16, P7, and P23 (Figure S5). These loci were all found on chromosomes 1 through 3, which are thought to possess loci strongly related to diapause life history timing based on previous selection experiments and phenotypic association studies [56,77,78,79,80]. Moreover, six of the seven loci resided in putative inverted regions in *R. pomonella* identified in Michel et al. [56]. Each of the other species-level taxa surveyed showed a complex relationship to the clinal variation present in the hawthorn race. None of the taxa mirrored the *R. pomonella* clines exactly nor were any of the taxa derived exclusively from a single region (northern or southern) of the distribution of hawthorn flies (Figure S5). Each of the species-level taxa therefore showed evidence for a locus-by-locus mosaic pattern similar to previous findings for the flowering dogwood fly, with a mixture of northern-like and southern-like variation at different loci [22]. Flowering dogwood fly populations were primarily “southern-like” in their genetic constitution relative to hawthorn-infesting flies (Figure S5). Of the seven microsatellites, only P23 was significantly more “northern-like” than the hawthorn race of *R. pomonella*, with a second locus (P70) showing a similar tendency but with a confidence interval overlapping the hawthorn mean (Figure S5). *Rhagoletis mendax* had mid-latitude like allele frequencies at three loci (P71, P37, and P80), significantly northern-like allele frequencies at three loci (P70, P7, and P23), and strongly southern-like allele frequencies at P16. *Rhagoletis zephyria* had consistently northern-like alleles at four loci (P37, P70, P16, and P7), consistently southern alleles at one locus (P80), mid latitude allele frequencies at one locus (P71), and a mix of northern and mid-latitude alleles at one locus (P23). The single populations of *R. cornivora* did not allow for a statistical test of clinal position. However, alleles at this population were generally northern for five out of the seven loci and similar to mid-latitude hawthorn allele frequencies at the remaining two loci. Overall, these findings are consistent with the hypothesis that life history variation adaptively maintained in the *R. pomonella* clines was co-opted during the radiation of the RPSG onto hosts with distinct fruiting phenologies. 

### 3.4. Patterns of Differentiation between Sympatric Taxa in the RPSG

**Figure 3:**
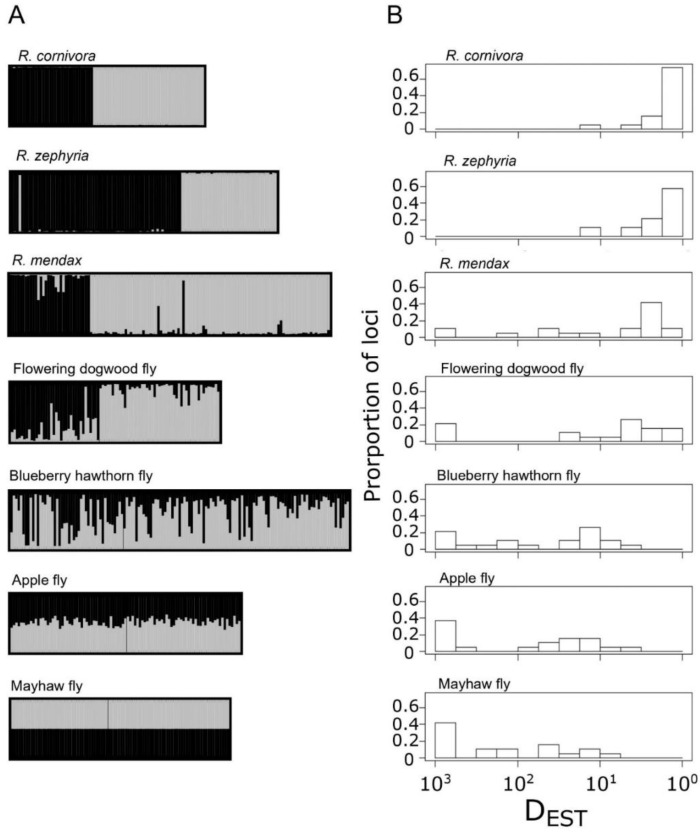
Patterns of genetic differentiation at paired sympatric comparisons between *R. pomonella* and taxa in the *R. pomonella* species group: (**A**) barplots of K = 2 structure analyses for each set of paired populations and (**B**) plots of the distribution locus-specific genetic differentiation for each paired population, measured as D_EST_.

Pairwise sympatric comparisons of derived complex members to *R. pomonella* revealed a transition from a best fit of K = 1 to K = 2 along the speciation continuum in the RPSG (Figure 3A; Table S7). Specifically, comparison of the host races within *R. pomonella*, including mayhaw, apple, and blueberry fly populations did not form discrete genotypic clusters. However, for the flowering dogwood fly, *R. mendax*, *R. zephyria*, and *R. cornivora* populations, the data strongly support a model of two largely independent populations at closely sympatric sites.

Patterns of locus-specific population genetic differentiation at each of the sympatric comparisons were largely heterogeneous for both host race and species-level comparisons (Figure 3B). Values of D_EST_ for the mayhaw, apple, and blueberry hawthorn populations ranged from 0.001 to 0.101, 0.001 to 0.281, and 0.001 to 0.240, respectively. Two of the species-level taxa, the flowering dogwood fly and *R. mendax*, also displayed some very weakly differentiated loci at these particular sympatric sites, with ranges of 0.001–0.603 and 0.001–0.870, respectively. Neither *R. zephyria* nor *R. cornivora* displayed any loci on the weakly differentiated left end of the scale, ranging from 0.078 to 0.922 and 0.078 to 1, respectively. The distribution of genetic differentiation for the three host race taxa follow a generally ‘L-shaped’ pattern of a high proportion of relatively undifferentiated loci combined with a long rightward tail of more differentiated loci, consistent with models of early stages of divergence along the speciation continuum (Figure 3B) [81,82]. The flowering dogwood fly and *R. mendax* both show a potentially transitional distribution, with a strong rightward shift in the bulk of the loci, with a handful of lingering undifferentiated loci (Figure 3B). The distributions for *R. zephyria* and *R. cornivora* showed a complete lack of weakly differentiated loci and were extremely similar (t = 1.402, *p* = 0.1696, *df* = 35), showing that an indistinguishable distribution of allelic differentiation can be reached for taxa with and without on-going gene flow.

**Figure 4:**
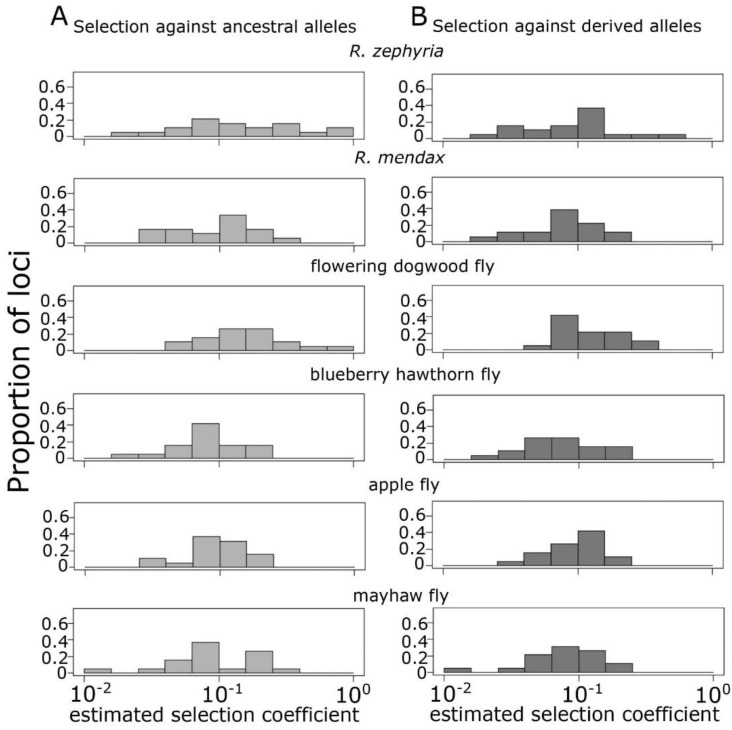
Distribution of estimated selection coefficients needed to maintain observed frequency differences between microsatellite allele classes under gene flow for each pairwise sympatric comparison. (**A**) Estimated selection coefficients against ancestral (hawthorn) allele in populations of the derived taxa and (**B**) estimated selection coefficients against allele favored in the derived population in the sympatric hawthorn population.

Selection coefficients necessary to maintain the observed allele frequency differences given estimated rates of gene flow between sympatric taxa showed similar distributions across most pairs of taxa (Figure 4). None of the six comparisons showed a significant bias toward stronger selection coefficients between the ancestral and derived populations (paired t-tests, *p* > 0.05 for each). Observed allele frequency differences between the mayhaw and green hawthorn population under an assumed gross migration rate of 5% can be maintained by an average selection coefficient of 0.114 (0.014–0.29) against green hawthorn alleles in mayhaw flies and 0.095 (0.014–0.2) in the reverse direction. For apple and downy hawthorn flies at 4.5% gene flow, the distribution of estimated selection coefficients were highly similar (mean = 0.105 for both and ranges of 0.034–0.181 and 0.035–0.199, respectively). Selection coefficients for blueberry hawthorn and mayhaw at 4% gene flow both averaged 0.094 with ranges of 0.021–0.199 and 0.018–0.244, respectively. Distributions were also similar for *R. mendax* and downy hawthorn, assuming 2% gene flow, averaging 0.115 (0.028–0.258) and 0.096 (0.024–0.226), respectively. However, the comparisons for sympatric hawthorn with flowering dogwood and *R. zephyria* both showed markedly stronger selection coefficients and considerable asymmetry across sympatric taxa. While the means selection coefficients did not differ, the variance was markedly higher in the derived compared to the ancestral taxa in both comparisons (F-tests, F_18,18_ = 8.67, *p* < 0.0001; F_18,18_ = 4.42, *p* = 0.0028, respectively). At a conservatively estimated 3% gene flow, selection against flowering dogwood alleles in downy hawthorn flies had a mean coefficient of 0.139 (0.059–0.339), while selection against downy hawthorn alleles in flowering dogwood flies averaged 0.219 (0.44–1.0). The value of 1 for section against hawthorn alleles in flowering dogwood for a single locus (P23) indicates that the observed (pooled) allele frequency could not be maintained in the face of 3% migration at equilibrium. 

The black hawthorn–*R. zephyria* comparison assumed a migration rate of 1%, consistent with observed hybridization rates in the Pacific Northwest [65]. Selection against *R. zephyria* alleles in black hawthorn flies averaged 0.131 (0.019–0.623), with 0.259 (0.019–1.0) for the reverse. In this case, two loci (P70 and P16) had allele frequency differences that were too high to be maintained by selection at 1% gene flow at equilibrium. Given the observed presence of hybrids between hawthorn and both the dogwood fly and *R. zephyria*, it is unlikely that the lack of fit of these extreme estimated coefficients to our model is the result of unrealistic migration rates. Instead, these results are likely due to the simplifications of symmetrical migration rates or lack of dominance effects. Nevertheless, these results indicate a role for very strong post-zygotic selection in maintaining genomic differentiation along the speciation continuum in the RPSG. 

## 4. Discussion

Species complexes involving on-going divergence in the face of gene flow among populations and taxa that differ in the magnitude of reproductive isolation and population genetic differentiation may offer unique windows into the evolutionary processes and genomic conditions underlying the formation of new biodiversity. While such systems do not represent a true chronoseries of events in a single speciation trajectory [83], comparative approaches in these systems may still offer insight into how the origin of species unfolds [19,22,84]. Here, we show that features of the natural history and population genetic relationships among the RPSG make it a particularly apt proxy for studying transitions along the speciation continuum. 

### 4.1. On-Going Gene Flow in the RPSG

Demonstrating active migration across diverging taxa is critical for interpreting genomic patterns of differentiation during incipient speciation. In the absence of gene flow, factors such as demographic history and within-population selection regimes have the potential to strongly shape patterns of heterogeneous differentiation between taxa [10,11,85]. However, when non-negligible migration is present, patterns of differentiation must be maintained under the homogenizing influence of gene flow and recombination across the genome. Thus, determining whether a particular system is useful for testing hypotheses about the genomics of speciation under the constraints imposed by active gene flow [86] requires establishing that migration is indeed occurring. Here, we confirm the existence of F1 and backcross individuals between *R. pomonella* and each of the named in-group taxa of the RPSG (Tables S3–S6, Figure S3). Genotypically scored hybrids were previously known for *R. zephyria* [65,87] and dogwood flies [22], but not for *R. mendax* [88]. For less diverged taxa in the RPSG, we are unable to apply this genotypic approach to identifying on-going gene flow due to the lack of strongly supported clustering in STRUCTURE. While gene flow between apple and downy hawthorn flies has been confirmed via mark–recapture studies [50], these laborious experiments have not been carried out between other taxa, like the blueberry hawthorn and mayhaw flies. For these populations of southern hawthorn flies, existing data strongly support on-going gene flow for two reasons. First, similar to the apple and downy hawthorn flies, blueberry hawthorn, mayhaw, and green hawthorn flies share all of their microsatellite allelic diversity. Second, prezygotic RI, based on divergence in eclosion timing and olfactory behavior appears to be incomplete among these taxa [36,37]. 

### 4.2. The Nested Nature of the RPSG Adaptive Radiation

Inferences from comparisons from different points along the speciation continuum may be stronger when the cases in questions have some common genomic underpinnings, ecological conditions, or biogeographic contexts. Species complexes where multiple derived members stem from the same pool of ancestral variation may be ideally suited for making direct comparisons about different stages of the speciation process. Here, we addressed the question of the relationship of the derived members of the RPSG to the putatively ancestral hawthorn-infesting populations of *R. pomonella* by examining microsatellite variation from three perspectives: the distribution of allelic diversity, population-level clustering, and how variation in derived populations reflects ancestral latitudinal clines in *R. pomonella*. The complex biogeographic history of *R. pomonella* coupled with the on-going gene flow and rapid nature of the RPSG radiation precludes a simple molecular systematic approach to defining relationships within the complex. The sequence variation segregating within the RPSG is marked by deeply divergent haplotypes, stemming from a history of repeated bouts of allopatry and secondary contact among ancestral populations of hawthorn-infesting populations of *R. pomonella* in the highlands of Mexico, starting ~1.5 mya [42,43]. This deep ancestral variation is shared among the derived members of the RPSG, and thus gene tree-based approaches are a poor fit for resolving relationships among this species complex [30]. 

Patterns of microsatellite variation within the RPSG are consistent with the in-group taxa representing a nested set of sibling taxa, likely each derived from hawthorn-infesting populations of *R. pomonella*. None of the in-group taxa showed fixed differences from *R. pomonella* at any of the 19 loci examined (Table 2). Both *R. mendax* and *R. zephyria* contained private alleles relative to *R. pomonella* at nine and five loci, respectively (Table 2), indicating that these more diverged taxa may be on the cusp of evolutionary independence. Otherwise, all the microsatellite variation within the in-group taxa represented a subset of the allelic variation present in the downy hawthorn-infesting *R. pomonella* (Table 2; Figure 1). 

In the population genetic distance network analysis, three of the in-group taxa, the flowering dogwood fly, *R. mendax*, and *R. zephyria*, form discrete genetic clusters across their geographic ranges, unlike *R. pomonella* (Figure 2). The clustering pattern for the three derived taxa may be indicative of more advanced stages along the speciation continuum, such that local gene flow from alternative host-associations begins to have a negligible effect on the population structure of these derived taxa across space [22,89,90]. For instance, despite documented, on-going hybridization, flowering dogwood fly populations have more similar allele frequencies to other flowering dogwood fly populations over 1000 km away than to closely sympatric hawthorn-infesting *R. pomonella* populations (Figure 2) with whom they are actively exchanging genes (Table S4). The patterns for *R. mendax* and *R. zephyria* are qualitatively similar but of an even greater magnitude (Figure 2). The *R. pomonella* populations in the network are largely arranged by latitude (Figure 2). While the positioning of the dogwood flies across this North–South gradient of *R. pomonella* was moderately well-supported (75% bootstrap support of clustering with a mid-latitude hawthorn population), the relationship of *R. mendax* and *R. zephyria* to that ancestral latitudinal variation was not well resolved in the network (Figure 2).

Our results suggest that the more divergent derived taxa in the RPSG may represent mosaics of latitudinal clinal variation segregating within ancestral *R. pomonella* populations, and this reshuffling of standing geographic variation may have been important to the overall radiation of the species group. Understanding adaptive latitudinal clines in *R. pomonella* was essential to clarifying the genetic relationship between the hawthorn and apple races [43,44,53,54,55,91]. Moreover, it is thought that this adaptively maintained variation in life history traits across latitude in the hawthorn population was co-opted to exploit a novel temporal niche in the formation of the apple race [42,53,56]. Phenological differences among host plants represent a major axis of ecological divergence in the radiation of the RPSG. Shifts in life history timing for univoltine insects like *R. pomonella* require adaptation to differing conditions both at the onset and termination of diapause, the eco-physiological dormancy phase that governs seasonal developmental timing for many insects [92]. In the case of the apple race of *R. pomonella*, this involves a complex set of gene by environment interactions with multiple pre- and post-winter thermal cues [93]. Studies on genetic associations with life history timing traits in *R. pomonella* suggest that the trait is extremely polygenic, involving widespread changes in gene expression [94,95] and statistical associations and response to selection experiments for over 100 independent genomic regions [78,80,95,96]. Thus, life history timing in *Rhagoletis* appears to be the sort of genetically complex trait that may be severely limited by the need for de novo mutations. 

Here, we found that allelic variation within each of the taxa was not drawn from single points along the hawthorn clines nor did any of the taxa completely mirror the clinal variation in the hawthorn race (Figure S5). Rather, like the apple race, variation within each of the taxa was drawn from different points along the ancestral clines for different loci (Figure S5). This finding is congruent with the hypothesis that the broad latitudinal clines in life history traits present in the hawthorn race provided a deep reservoir of standing genetic variation that could be rearranged (via gene flow across the clines) to eventually form multilocus genotypes adaptive to novel temporal niches during the radiation of the RPSG. The clinal variation in *R. pomonella* provides one example of how standing genetic variation that proves to be adaptive to yet unutilized niches may be maintained in ancestral populations. Species with broad geographic ranges that exploit ephemeral resources may possess considerable genetic variation for several different aspects of thermal and seasonal adaptation. This complex clinal variation may be reshuffled through gene flow to produce genotypes that allow for the colonization of novel multidimensional phenological niches. Scenarios that allow this kind of cooption of pre-existing coadapted gene complexes fit well with recent models of ‘combinatorial’ speciation [97].

### 4.3. The Build-Up of Genetic Differentiation in the RPSG

The taxa in the RPSG represent a broad range of levels of divergence, from host races, or even less differentiated ‘host forms’ (*sensu* Funk [98]) in the case of very recently evolved host-associated differentiation in the Pacific Northwest [48], through to well-differentiated species with fixed genetic differences. This variation in divergence, combined with the ecology and biogeography of these flies and the established on-going gene flow and nested relationships based on shared allelic variation from above, makes the *R. pomonella* system a particularly apt surrogate for transitions along the speciation continuum. Thus, these flies present a nearly ideal system for understanding the interaction of selection, migration, recombination, genetic variation, and genomic architecture during speciation with gene flow. We do not propose that the relatively coarse-grained microsatellite data in the current study represent a definitive test of any specific models of genomic divergence during speciation with gene flow, per se. However, patterns of population genetic differentiation in the RPSG documented here do speak to how speciation may have proceeded in these flies and can be compared to predictions of models of speciation with gene flow. 

**Figure 5:**
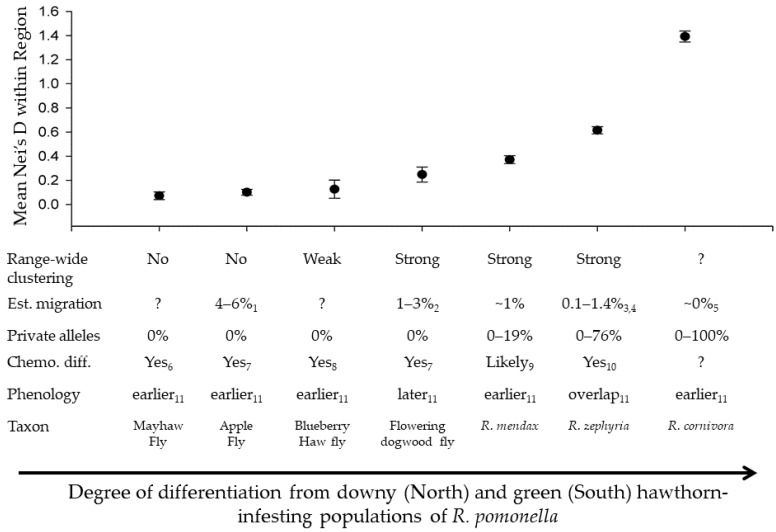
Summary of genetic and phenotypic differentiation in the *R. pomonella* species complex, including mean Nei’s D between each population of that taxon and each hawthorn-race *R. pomonella* within the respective region, the presence of range-wide clustering in population genetic distance networks, estimated migration from previous studies, the range of frequencies for alleles private to each taxon relative to *R. pomonella*, the presence of evidence for chemosensory behavioral differentiation from previous studies, and the relationship of host plant phenology to that of co-occurring *R. pomonella*. Subscripts denote the reference from which data was extracted: 1 = [50]; 2 = [22]; 3 = [87]; 4 = [88]_;_ 5 = [117]; 6 = [118]; 7 = [33]; 8= [119]; 9 = Linn CE, personal communication; 10 = [29]; 11 = [120].

The taxa in the earlier host race portion of the divergence continuum in the RPSG show strong phenotypic differentiation in traits directly related to both host plant adaptation and reproductive isolation [33,34,35,36,37] but relatively weak overall genetic differentiation (Figure 3 and Figure 5). None of these taxa display any private alleles, and thus share essentially the same pool of allelic variation (Table 2). They do not form discrete genotypic clusters at either the population or individual levels (Figure 2 and Figure 3A). Moreover, their genomic distribution of differentiation is characteristically ‘L shaped’, with most loci showing modest differentiation with a long right tail of more differentiated loci (Figure 3B). The partial differentiation observed at this end of the continuum in the RPSG may represent stalled cases of ecological divergence [99], with populations at a (potentially long-term) balance of divergent selection, migration, and available genetic variation. 

The flowering dogwood fly similarly shares all of its microsatellite allelic variation with *R. pomonella* (Table 2). However, this taxon represents a distinct shift along the speciation continuum that may be indicative of a critical transitional phase by forming a genetic cluster across its geographic range (Figure 2) [22]. The pattern of individual-level clustering and the genomic distribution of microsatellite differentiation are markedly shifted as well (Figure 3). In a previous study, simulation modeling based on estimates of gross migration and observed allele frequency differences for the apple race of *R. pomonella* and the flowering dogwood fly suggest that this shift was due to a modest increase in divergent selection resulting in a global reduction in effective migration for the flowering dogwood fly [22]. This observation of a qualitatively different clustering pattern emerging from a subtle, quantitatively continuous increase in divergent selection may be the result of the dogwood fly approaching or reaching a threshold for ‘tipping points’ in the emergence of evolutionary independence along the speciation continuum [81,82,100]. 

The other two in-group taxa, *R. mendax* and *R. zephyria* showed even stronger patterns of clustering (higher bootstrap support and longer branch lengths) than the dogwood fly (Figure 2) as well as further rightward shifts in their distributions of genic differentiation (Figure 3B). The presence of on-going gene flow in both of these taxa demonstrates how complete reproductive isolation makes for a poor criterion in species concepts, as very strong genetic discontinuities are able to persist despite regular hybridization [101]. Moreover, the population genetic differentiation between *R. pomonella* and *R. zephyria* is strikingly similar to that of *R. cornivora*, a taxon that shows fixed mtDNA and morphological differences from *R. pomonella* [30] (Figure 3B). Both taxa possess private alleles at the majority of loci (Table 2), and their distributions of locus-specific differentiation were not statistically different (Figure 3B). Thus, a state of strong differentiation comparable to that of a reciprocally monophyletic taxon can be reached even in the face of on-going gene flow. It is important to note that our conclusions about the strength of distribution of differentiation for *R. cornivora* are limited to variation within the single population samples. This taxon has a wide geographic distribution across eastern North America [24] (Figure S1), and therefore broader sampling of *R. cornivora* may reveal even stronger patterns of allele frequency differentiation or more genomically homogenous topology of differentiation. 

In the face of strong, continuous gene flow with reasonably large effective population sizes, allele frequency differences between diverging populations must be maintained by selection countering the homogenizing effects of gross migration [102]. Thus, outlier-based analyses of loci under divergent selection that rely on an assumption that ‘baseline’ differentiation is the result of neutral drift/migration balance may often fail to detect even strong selection when differentiation extends beyond a handful of genomic regions in cases with documented gene flow [22,56]. The genomic architecture of traits underlying divergent selection likely has important consequences of how divergence and speciation proceed [103]. Our analysis of the selection coefficients needed to maintain the observed allele frequency differences in a two-island model of selection/migration equilibrium suggests that that transition to further positions along the speciation continuum may be driven more by increases in the genome-wide effects of selection rather than increased strength of localized selection (Figure 4). While strong localized selection may be present at intermediate stages of divergence, such as locus P23 in flowering dogwood fly (Figure 4), more differentiated taxa showed a general rightward shift of the overall distribution of estimated selection coefficients. In our simplified analytical model, we examined the potential for direct selection acting on independent loci for driving the observed allele frequency differences. In reality, the allele frequency differences in these microsatellite loci are likely maintained in the face of gene flow by the combination of indirect selection at linked loci and genome-wide effects [102], with our estimates representing the cumulative strengths of these factors at each locus. Previous studies have shown that one of the key traits under divergent selection in this system, diapaus regulation, is highly polygenic in *R. pomonella*, with low to moderate effect loci distributed across the genome [56,77,78,80,95,96] with similar patterns for *R. mendax* [104]. This variation is also associated with complex inversion polymorphisms that reduce recombination across much of chromosomes 1, 2, and 3 [56,79,80,91], making it not unlikely that some of our microsatellite markers are indeed linked to loci under selection. However, such multifaceted selection is also expected to produce genome-wide effects of divergent selection to reduce effective migration of unlinked loci as well [81,100]. Thus, the pattern of overall increases in the strength of selection across all loci is consistent with a transition from genic to genome-wide processes affecting selection against migrants, hybrids, and backcrosses as differentiation proceeds along the speciation continuum [100].

Whether these patterns are peculiar to *Rhagoletis* or are indicative more broadly of the process of speciation with gene flow remains to be seen. However, genetic and phenotypic evidence from two other insect speciation systems suggest a potential role for abrupt shifts resulting from the continuous build-up of differentiation. Peccoud et al. [19] found that for 11 host-associated ‘biotypes’ of pea aphids in Europe pairwise genetic distance was generally continuous but that observed hybridization was markedly discontinuous between host races and species. A comprehensive review of the *Heliconius* system by Merrill et al. [105] notes that despite a generally continuous pattern of the strength of reproductive isolation between sub-species and species in *Heliconius* and observed gene flow in both, genomic evidence suggests that the transition from sub-species to species may be characterized by a shift from localized to widespread genomic differentiation [106,107,108]. Both systems suggest the potential for non-linear transitions along the speciation continuum emerging from the continuous build-up of differentiation. 

An important remaining question is what factors determine each taxon’s position along the speciation continuum. Why do some divergently specialized taxa persist in a state of weak differentiation while others have not? Are long-term host races the result of intrinsic limitations, such as exhausting standing genetic variation, or extrinsic limitations involving the selection pressures imposed by the derived host plant? Such questions are important considerations for many speciation systems, as we cannot be certain that any cases of partial reproductive isolation will ever progress further along the continuum [99,109]. For the apple race, time seems to be an obvious answer, as it first emerged less than 170 years ago [24,110]. However, the same cannot be said for the southern hawthorn-infesting host races, which are quite similar to the apple race in terms of phenotypic and genetic differentiation [37]. One possibility is that additional axes of ecological adaptation are needed to push taxa into species-level divergence. For instance, larval feeding physiology does not differ between the downy hawthorn and apple races of *R. pomonella* [111], but nutritional or secondary compound differences among the other host plants may be an important component of adaptation elsewhere in the complex. Feeding performance is certainly important in other cases of ecological speciation [112,113,114,115], and this may be the case of the *RPSG* as well. Larval transplant experiments showed differences in host-specific performance between *R. pomonella* and *R. zephyria* [116]. Comparative studies of speciation with gene flow cannot rely on genome scans alone. Rather, understanding how speciation proceeds along the continuum also requires thorough investigations of how the ecological context of divergence may differ for the populations being compared. 

## 5. Conclusions

Our results indicate that the RPSG represents a nearly ideal system for testing hypotheses about how ecological and genomic divergence proceeds along the speciation continuum in the face of gene flow. Four factors set the stage for these flies providing a powerful window into the origin of biodiversity via ecological adaptation: (1) the primary axes of divergence and reproductive isolation for each case involve the same facets of host-plant adaptation [29,34,36,37], (2) each of the in-group taxa are diverging in the face of active gene flow with ancestral hawthorn *R. pomonella* populations (Tables S3–S5), (3) the taxa likely represent a nested radiation from the same proximate ancestral variation (Figure 2 and Figure S5), and (4) the taxa span a range of hypothesized stages along the speciation continuum (Figure 3, Figure 4 and Figure 5). The RPSG can be divided into two phases of speciation, ‘pre-clustering’ and ‘post-clustering’ with the possibility of a transitional position for the dogwood fly (Figure 2 and Figure 3A). This shift to range-wide clustering is also associated with a shift in the distribution of genic differentiation from a state predominated by generally weak genome-wide differentiation with a handful of more differentiated genes to a state characterized by strong genome-wide differentiation (Figure 3B). These two distinct phases bolster the idea that the shift of reproductive isolation from a property of specific loci to a property of the entire genome is a critical transition in speciation.

The microsatellite data presented here are too coarse-grained to adequately represent the dynamics of diverging genomes. The obvious extension of this work in the RPSG will be to compare the genomic topology of divergence among these taxa using high-coverage SNP data. These data will almost certainly bring additional insights into how ecology, genetic variation, and genome structure interact in the origin of species and allow for more rigorous tests of theoretical predictions of how genomic differentiation proceeds in the face of gene flow. One important consideration for future studies in the RPSG will be to combine population genomic analyses with functional genomics of the traits under divergent selection. Our results here also highlight how the most powerful systems for understanding speciation may be those for which it is still an on-going process. This requires taking an explicitly population-based approach (as opposed to making comparisons based on a handful of sequenced individuals) in systems that are best described by population genetic distance rather than fixed molecular genetic distances and post-speciation lineage sorting. 

## Figures and Tables

**Table 1:** Host plant origin, numerical designation, location, latitude [N] and longitude [W] in degrees, year sampled, and number of individuals genotyped (*n*). Subscript letters in the population number column designate sympatric comparisons.

Taxa	Host	#	Location	Lat., Long.	Year	*n*
*R. pomonella*	Hawthorn (*Crataegus mollis*)	1_a_	Newaygo Co., MI	43.35, 85.9	2002	48
		2_b_	Allegan Co., MI	42.6, 86.15	2002	96
		3_d_	Cass Co., MI	41.88, 86.23	2002	49
		4_c_	Champaign Co., IL	40.08, 88.23	2002	46
		5	New Madrid Co., MO	36.53, 89.43	2008	41
	Hawthorn (*C. viridis*)	6	Caldwell Pr., LA	32.04, 91.55	2007	48
		7_e_	La Salle Pr. LA	31.27, 92.07	2009	56
		8	Fort Bend Co., TX	29.22, 95.36	2007	98
	Hawthorn (*C. opaca*)	9_f_	Nacogdoches Co., TX	31.31, 94.46	2006	94
		10_e_	La Salle Pr. LA	31.27, 92.07	2009	40
	Hawthorn (*C. brachyacantha*)	11_e_	Nacogdoches Co., TX	31.31, 94.46	2008	47
		12_f_	Angelina Co., TX	31.21, 94.45	2010	49
	Hawthorn (*C. douglassii*)	13	Clark Co, WA	45.7, 122.63	2009	39
	Hawthorn (*C. douglassii*)	14_g_	Skamania Co., WA	45.6, 122.11	2009	47
	Apple (eastern)	15_a_	Newaygo Co., MI	43.35, 85.9	2002	47
		16	Allegan Co., MI	42.6, 86.15	2002	96
		17	Cass Co., MI	41.88, 86.23	2002	47
		18	Champaign Co., IL	40.08, 88.23	2002	48
	Apple (western)	19	Clark Co., WA	45.7, 122.63	2009	48
		20	Skamania Co., WA	45.6, 122.11	2009	105
Flowering Dogwood fly	Dogwood (*Cornus florida*)	21_d_	Cass Co., MI	42.0, 85.97	2006	36
		22	Lake Co., TN	36.45, 89.3	2010	40
		23	Nacogdoches Co., TX	31.31, 94.46	2007	39
*R. mendax*	Blueberry (*Vaccinium corybosum*)	24_b_	Allegan Co., MI	42.6, 86.15	2007	32
		25	Burlington Co., NJ	39.82, 74.53	2006	32
	Deerberry (*V. stamineum*)	26	Aiken Co., SC	33.54, 81.49	2009	26
		27	Richland Co., SC	34.08, 80.90	2009	32
*R. zephyria*	Snowberry (*Symphiocarpos albus*)	28	Clark Co., WA	45.73, 122.63	2009	48
		29_g_	Skamania Co., WA	45.6, 122.11	2009	72
		30	Etobicoke, ON	43.58, 79.54	2011	59
	Snowberry (*S. occidentalis*)	31	Musselshell Co., MT	46.57, 107.94	1994	29
		32	Polk Co., MN	47.52, 96.28	1994	29
*R. cornivora*	Silky Dogwood (*Cornus amomum*)	33_c_	Champaign Co., IL	40.08, 88.23	2001	34

**Table 2:** Frequency of private alleles for 19 microsatellites in four species-level taxa in the RPSC relative to *R. pomonella*. Frequency values represent the sum of allele frequencies for individual private fragment length polymorphism across all individuals in each taxon. For in-group taxa, designated private alleles were detected in more than one population of that taxon and absent in all other populations. This requirement of ≥2 populations could not be met in *R. cornivora*, due to a single population being sampled.

		Taxa			
LG	Locus	Dogwood Fly	*R. mendax*	*R. zephyria*	*R. cornivora*
1	p71	-	0.076	-	0.219
1	p37	-	-	0.084	-
1	p4	-	0.104	-	0.656
1	p3	-	-	0.236	1.000
2	p46	-	-	0.091	0.750
2	p73	-	-	0.036	0.241
2	p70	-	0.034	-	0.339
2	p80	-	-	0.051	-
3	p7	-	0.187	0.746	0.167
3	p16	-	-	-	0.353
3	p23	-	-	0.402	0.303
3	p66	-	-	-	-
4	p11	-	0.027	-	-
4	p29	-	-	-	0.912
4	p50	-	-	-	-
4	p60	-	-	-	0.054
5	p18	-	-	0.124	0.594
5	p9	-	-	-	1.000
5	p27	-	-	0.137	0.375

## Data Availability

An Excel spreadsheet of allele frequencies is included in the online supplementary material, and “Convert” format text files of individual genotype data will be submitted to Data Dryad upon publication.

## Supplementary Materials

The following supporting information can be downloaded at: https://www.mdpi.com/article/10.3390/genes13020275/s1, Figure S1. Range maps for RPSG taxa, based on host plant distributions from the USDA Plants database and fly collection records from previous studies: (A) downy hawthorn and green hawthorn; (B) apple host race of *R. pomonella* [55], newly introduced populations of *R. pomonella* in the Pacific Northwest [121], and blueberry hawthorn; (C) *R. mendax* [122]; (D) flowering dogwood; (E) *R. zephyria* [123] and western mayhaw [37]; (F) silky dogwood, host of *R. cornivora*. Figure S2. Collection sites for populations of flies genotyped and listed in Table 1. Open squares = hawthorn-infesting *R. pomonella*; closed squares = apple-infesting *R. pomonella*; open circles = *R. zephyria*; closed circles = *R. mendax*; pentagons = flowering dogwood fly, and star = *R. cornivora*. Figure S3. STRUCTURE bar plots for migrant/hybrid detection runs for *R. pomonella* species complex members divided by geographic region: (A) Northeast, (B) South, and (C) Pacific Northwest. Asterisks indicate individuals with posterior probabilities favoring migrant or hybrid origin. *** = parental migrant; ** = F1 hybrid; * = backcross. Figure S4. Neighbor-joining network based on Nei’s D (1972) estimated from microsatellite allele frequencies for 19 loci. Open squares = hawthorn-infesting *R. pomonella*; closed squares = apple-infesting *R. pomonella*; open circles = *R. zephyria*; closed circles = *R. mendax*; pentagons = flowering dogwood fly, and star = *R. cornivora*. Bootstrap values come from 10,000 replicates across loci. Numbers within nodes refer to sites listed in Table 1 and Figure S2. Figure S5. Principal component (PC1) scores of allele frequencies representing major clinal patterns within hawthorn-infesting *R. pomonella* populations (filled circles) for *R. mendax* (inverted triangles), the flowering dogwood fly (open circles), the apple race of *R. pomonella* (open squares), R. zephyria (open triangles), and *R. cornivora* (filled square) for seven major clinal loci on linkage groups 1 (A), 2 (B), and 3 (C). R2 values reflect linear regression of hawthorn-race PC1 versus latitude. Capital letters along the x-axis designate taxa that were significantly northern-like (N) or southern-like (S) at that locus for across all populations, based lack of overlap and directionality of 95% confidence interval with estimated mean of PC1 between the taxa and the hawthorn race of *R. pomonella*. Table S1. List of 19 microsatellite markers used in this study. Table S3. Count of putative parental migrants (P), F1 hybrids, and backcrosses (BX) among taxa of *R. pomonella* species complex members in the Northeastern United States. Counts represent individuals with the highest posterior probability of belonging to one of the migrant or hybrid classes as determined by STRUCTURE analysis. Receiving populations are listed in rows while source populations of migrants are listed in columns. Table S2. Mean inbreeding coefficient (F_IS_) across loci with 95% confidence intervals for each population, designations following Table 1. Table S3. Results of STRUCTURE runs establishing the presence of strong clustering patterns justifying subsequent migrant identification analyses, including mean Ln Likelihood, standard deviation, and ΔK across five replicates, each from 750,000 iterations following a burn-in period of 500,000 iterations, of six values of K surrounding Likelihood plateaus identified from an initial screen of K=1 – K = total number site/host combinations of five replicate runs of 500,000 iterations following burn-in periods of 250,000 for each of the three regional analyses. Table S4. Count of putative parental migrants (P), F1 hybrids, and backcrosses (BX) among taxa *of R. pomonella* species complex members in the Northeastern United States. Counts represent individuals with the highest posterior probability of belonging to one of the migrant or hybrid classes as determined by STRUCTURE analysis. Receiving populations are listed in rows while source populations of migrants are listed in columns. Table S5. Count of putative parental migrants (P), F1 hybrids, and backcrosses (BX) among taxa of *R. pomonella* species complex members in the Southeastern United States. Counts represent individuals with the highest posterior probability of belonging to one of the migrant or hybrid classes as determined by STRUCTURE analysis. Receiving populations are listed in rows while source populations of migrants are listed in columns. Table S6. Count of putative parental migrants (P), F1 hybrids, and backcrosses (BX) among taxa of *R. pomonella* species complex members in the Pacific Northwest of the United States. Counts represent individuals with the highest posterior probability of belonging to one of the migrant or hybrid classes as determined by STRUCTURE analysis. Receiving populations are listed in rows while source populations of migrants are listed in columns. Table S7. Mean estimated Ln likelihood and standard deviation across ten replicates of STRUCTURE analysis of *R. pomonella* species complex taxa and paired sympatric *R. pomonella* populations a for K = 1 and K = 2, using a burn-in of 500,000 followed by 1,000,000 MCMC repetitions under a correlated allele frequency with admixture model. “Δ Ln Lik” reports the change in mean Ln likelihood between K = 2 and K = 1.

## Appendix A

### Appendix A.1. Microsatellite Genotyping Methods

Genomic DNA was isolated from whole flies (in either adult or pupal form) using PUREGENE extraction kits (Qiagen, Hilden, Germany). Locus-specific olignucleotide primers were used to amplify microsatellites for 35 cycles under the following conditions: (initial denaturing of 94 °C for 60 s) 35 X (94 °C for 20 s, 58 °C for 15 s, 72 °C for 30 s) (final elongation incubation of 72 °C for 10 min). The annealing temperature of some loci differed from the 58 °C used for most, specific annealing temperatures for these loci can be found in the supporting documentation of Michel et al. (2010) [56]. Genotyping was performed via capillary electrophoresis using a Beckman-Coulter CEQ8000. PCR fragment sizes were scored manually using Beckman-Coulter’s Fragment Analysis software. New data (for *R. mendax*, *R. zephyria*, and *R. cornivora*) were combined with data from previously published data on *R. pomonella* and the flowering dogwood fly as raw estimated fragment sizes before being binned into called allele classes.

Allele calls were made by manually re-binning this combined dataset of raw fragment sizes. Estimated fragment sizes were generally normally distributed within Beckmann-Coulter’s default bin width of 0.85 bp. As with previous *Rhagoletis* microsatellite studies, many loci contained alleles with 1bp phase shifts. This is due to the combined factors of biogeographic history (deeply divergent inversion polymorphisms assorting in the population; [44]) and the complex nature of some of the repeat motifs [63]. Individual flies that were re-genotyped as positive controls across the collection of the RPSC microsatellite database (*n* = 30) had a mean shift in raw estimated fragment length of 0.09 bp ± 0.025 SE.

### Appendix A.2. Detection of Putative Migrants and Hybrids

Previous studies focused on particular members of the *R. pomonella* species group have detected putative migrants, F1 hybrids, and backcross individuals from microsatellite genotype data [22,65]. Here, we expanded that approach across the entire dataset to test for the presence of genotypes among all host associations and taxa consistent with on-going gene flow. A full description of these methods can be found in the online supplementary material. Briefly, our approach involved using STRUCTURE 2.3.4 to infer the total number of discrete genotypic clusters within broad geographic regions (North, South, and Pacific Northwest), and then using these clusters as a priori population designations for the STRUCTURE’s built-in function for detecting putative migrants, hybrids, and backcrosses. For each regional hybrid detection analysis, we conducted three replicate analyses under the admixture with correlated allele frequencies model at each of three values of migration prior (MIGPRIOR): the default 0.05, 0.01, and 0.1. Each analysis was run for 1,000,000 iterations after discarding a burn-in of 500,000 iterations. These options in STRUCTURE produce an output of individuals categorized as belonging to one of four genotypic classes based on posterior probabilities: pure natal-host genotypes, pure parental migrants possessing non-natal host genotypes, F1 hybrids having non-natal host heritage one generation back, or backcrosses having non-natal host heritage two generations back. Alongside these multiway hybrid detection analyses, we also run each pairwise combination of complex members within regions separately.

### Appendix A.3. Allelic Richness Estimation

To quantify and compare levels of allelic richness among members of the *R. pomonella* species complex, allelic richness for each population was estimated using a rarefaction approach implemented in ADZE [68]. Populations were resampled up to their actual sample size in ADZE. We then fit the resulting rarefaction curves to a 2-parameter asymptotic function, forced through the origin, using a nonlinear regression function in R 2.13.1 (R Development Core, Vienna, Austria), to estimate the total number of alleles segregating in each population, regardless of sample size (in all populations this 2-parameter model proved to be a better fit for the data than a Michelis-Menten asymptotic function). Allelic richness estimates were then compared among taxa with at least three representative populations using one-way ANOVA with taxon as a factor in R, followed by a Tukey’s post hoc test.

### Appendix A.4. Clinal Variation

Evidence from previous studies suggests that the latitudinal clines associated with life-history timing in the hawthorn race of *R. pomonella* have played an important role in the origin of the apple race [53,56] and flowering dogwood fly [22] and that there is a strong signal of clinal variation within the radiation of southern hawthorn-infesting host races of *R. pomonella* [37]. In order to understand how the radiation of the wider *R. pomonella* species complex fits into this ancestral clinal variation, we compared the allele frequencies of the apple race, flowering dogwood fly, *R. mendax*, *R. zephyria*, and *R. cornivora* to the major patterns of latitudinal variation within the hawthorn race. We did this by first collapsing the variation in loci (many of which were highly polymorphic, with >40 observed alleles) within the putatively ancestral hawthorn flies using principal components analysis (PCA) and then comparing the derived taxa in the species complex to the major clinal variation within the hawthorn race. We took seven hawthorn-infesting populations of *R. pomonella* spanning a latitudinal transect of ~1400 km in the Midwestern United States, and performed PCA on the allele frequency variation at each locus using the function *prcomp* in R. We then performed a linear regression on the first principal component (to conservatively restrict the analysis to only the loci for which the major patterns of allelic variation were related to latitude) for each locus against latitude. Loci that resulted in a significant relationship between PC1 and latitude were designated as ‘major clinal loci’ and retained for further analysis. Allele frequencies for these loci were then collapsed in the other members of the species complex using the loading vector for each respective PC1of within-hawthorn variation. The resulting PC1-scores for each population were then plotted alongside the hawthorn flies to give a visual depiction of the relationships. We then scored the flowering dogwood fly, *R. mendax*, and *R. zephyria* as being northern-like or southern-like for each clinal locus depending on whether or not the mean PC1 score within each taxa was greater or less than zero and whether the 95% confidence interval of the mean (assuming a normal distribution) overlapped zero.

### Appendix A.5. Estimating Selection Coefficients

To understand the interaction of evolutionary forces in driving patterns of differentiation in these sympatric population pairs, we estimated the selection coefficients (*s*) needed to maintain the observed allele frequency differences given the estimated gross migration rates between taxa at equilibrium. Allelic variation in our highly polymorphic microsatellite loci was simplified in order to consider the fate of just two composite allele classe. For each comparison and each locus, we pooled the alleles that were more common in *R. pomonella* and the derived RPSG taxon, respectively. We then determined the expected change in the frequency of these two allele classes in each generation due to migration and asked what strength of selection against migrant alleles was needed to maintain the observed allele frequency difference at equilibrium. Modelling was performed using *Mathematica* 13.0 (Wolfram Research, Inc., Champaign, IL). Our two-island model assumed symmetric migration and equal population size (with no influence of drift). Using the designations *Rp* and *D* to indicate allele frequencies in the ancestral *R. pomonella* and derived populations in each pair, respectively, and *p* as the allele class favored in *R. pomonella* we model the expected change in *p* in the *R. pomonella* population due to migration as:

(A1) $$\Delta p_{Rp}=m\left(p_{D}-p_{Rp}\right)$$

Additionally, the equilibrium between migration and selection against alleles favored in the derived host population as:

(A2) $$0=\Delta p_{Rp}+\frac{p_{Rp}}{\bar{\omega }}\left(p_{Rp}\omega_{AA}+q_{Rp}\omega_{Aa}-\bar{\omega }\right)$$

where

(A3) $$\omega_{AA}=1\;\omega_{Aa}=1-sh\;\omega_{aa}=1-s$$

such that

(A4) $$0=\Delta p_{Rp}+\frac{p_{Rp}}{\left(p_{Rp}^{2}+2p_{Rp}q_{Rp}\left(1-hs\right)+q_{Rp}^{2}\left(1-s\right)\right)}\left(p_{Rp}+q_{Rp}\left(1-hs\right)-\left(p_{Rp}^{2}+2p_{Rp}q_{Rp}\left(1-hs\right)+q_{Rp}^{2}\left(1-s\right)\right)\right)$$

For the case of *h* = 0.5, solving for *s*:

(A5) $$s=\frac{2\Delta p_{Rp}}{\left(p_{Rp}-1\right)\left(2\Delta p_{Rp}-p_{Rp}\right)}$$

We used conservative estimates of migration rates for each population pair, based on mark–recapture data for apple/hawthorn [50] and genotypically detected hybridization rates from previously published data and this paper [22,54,65] for the dogwood, *R. mendax*, and *R. zephyria* comparisons. For the other comparisons that could not support STRUCTURE-based detection of migration (due to insufficient a priori clustering strength), we chose values on either side of the measured apple/hawthorn rate keeping in rank order with the overall strength of differentiation for those taxa. We report results for the following estimates of *m*: 5%, 4.5%, 4%, 3%, 2%, and 1% for mayhaw, apple, blueberry hawthorn, dogwood, *R. mendax*, and *R. zephyria*, respectively.
