# Supplementary material for: The Build-Up of Population Genetic Divergence along the Speciation Continuum during a Recent Adaptive Radiation of Rhagoletis Flies
Source: Genes (Basel). 2022 Jan 30;13(2):275. doi: 10.3390/genes13020275 (PMC8872456; doi:10.3390/genes13020275)
Supplement: Supplementary file 1 [file genes-13-00275-s001.zip › genes-1557809-supplementary.pdf]

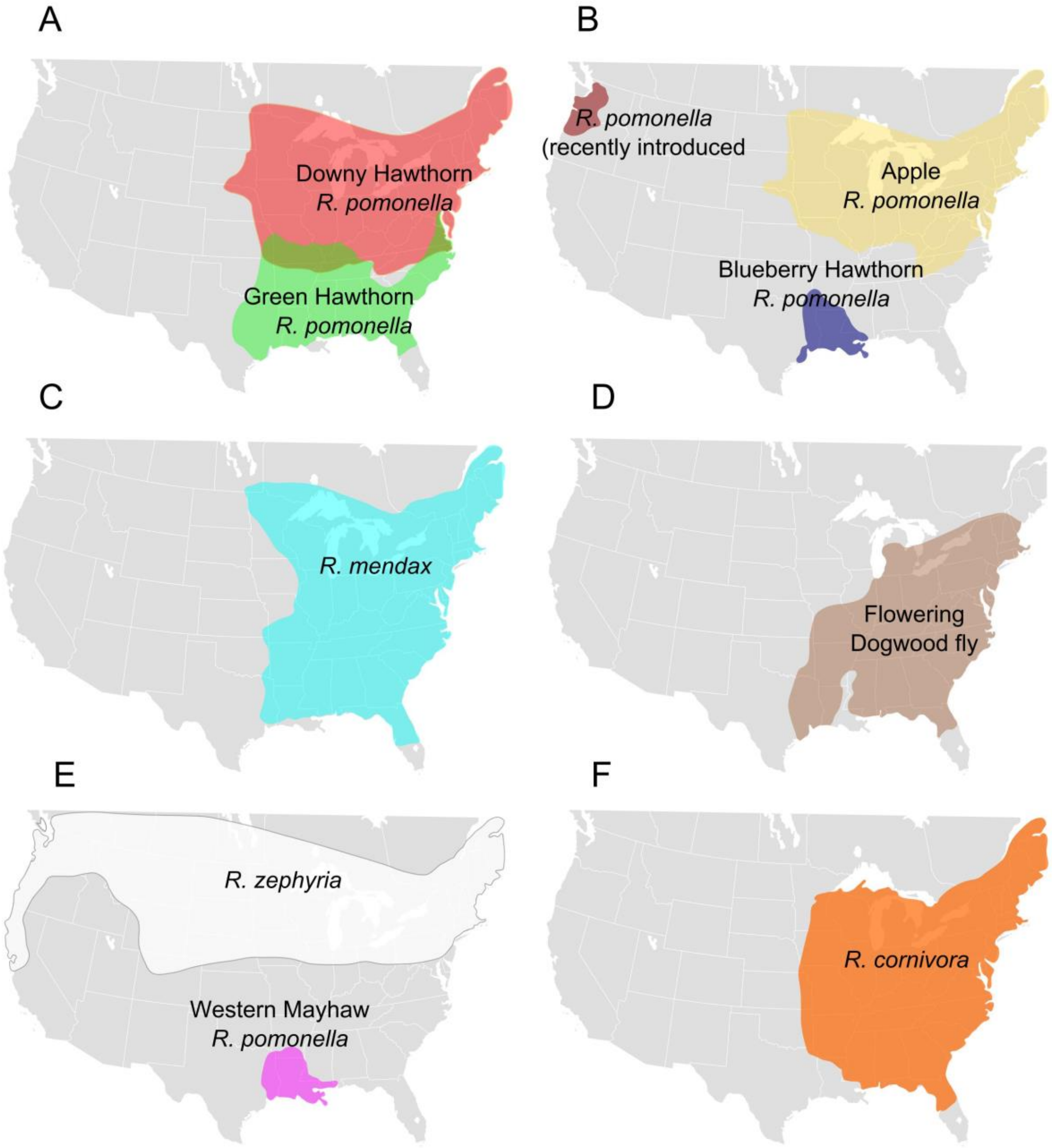

Figure S1. Range maps for RPSG taxa, based on host plant distributions from the USDA Plants database and fly collection records from previous studies: (A) downy hawthorn and green hawthorn; (B) apple host race of *R. pomonella* [55], newly introduced populations of *R. pomonella* in the Pacific Northwest [122], and blueberry hawthorn; (C) *R. mendax* [121]; (D) flowering dogwood; (E) *R. zephyria* [123] and western mayhaw [37]; (F) silky dogwood, host of *R. cornivora*.

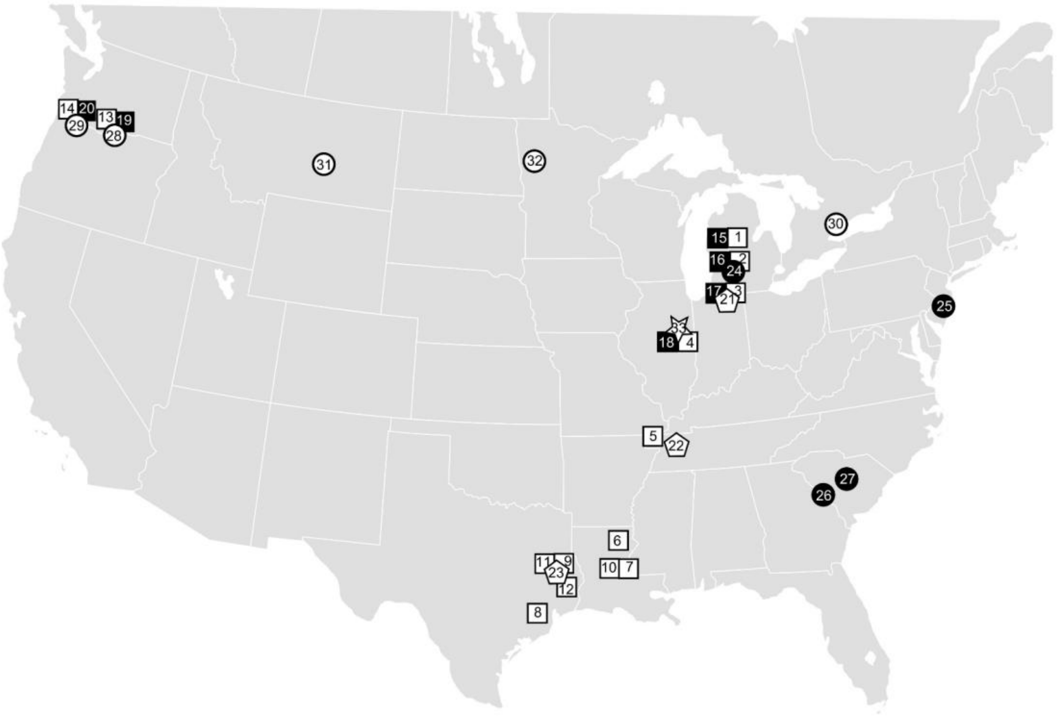

Figure S2. Collection sites for populations of flies genotyped and listed in Table 1. Open squares = hawthorn-infesting *R. pomonella*; closed squares = apple-infesting *R. pomonella*; open circles = *R. zephyria*; closed circles = *R. mendax*; pentagons = flowering dogwood fly, and star = *R. cornivora*.

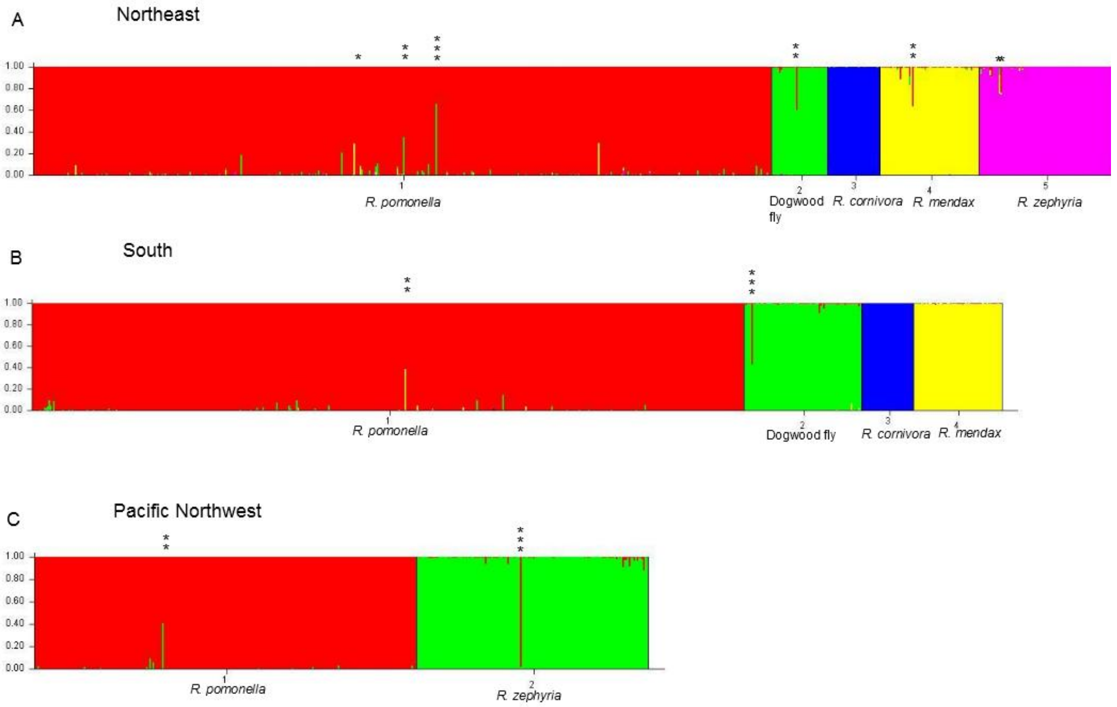

Figure S3. STRUCTURE bar plots for migrant/hybrid detection runs for *R. pomonella* species complex members divided by geographic region: A) Northeast, B) South, and C) Pacific Northwest. Asterisks indicate individuals with posterior probabilities favoring migrant or hybrid origin. *** = parental migrant; ** = F1 hybrid; * = backcross.

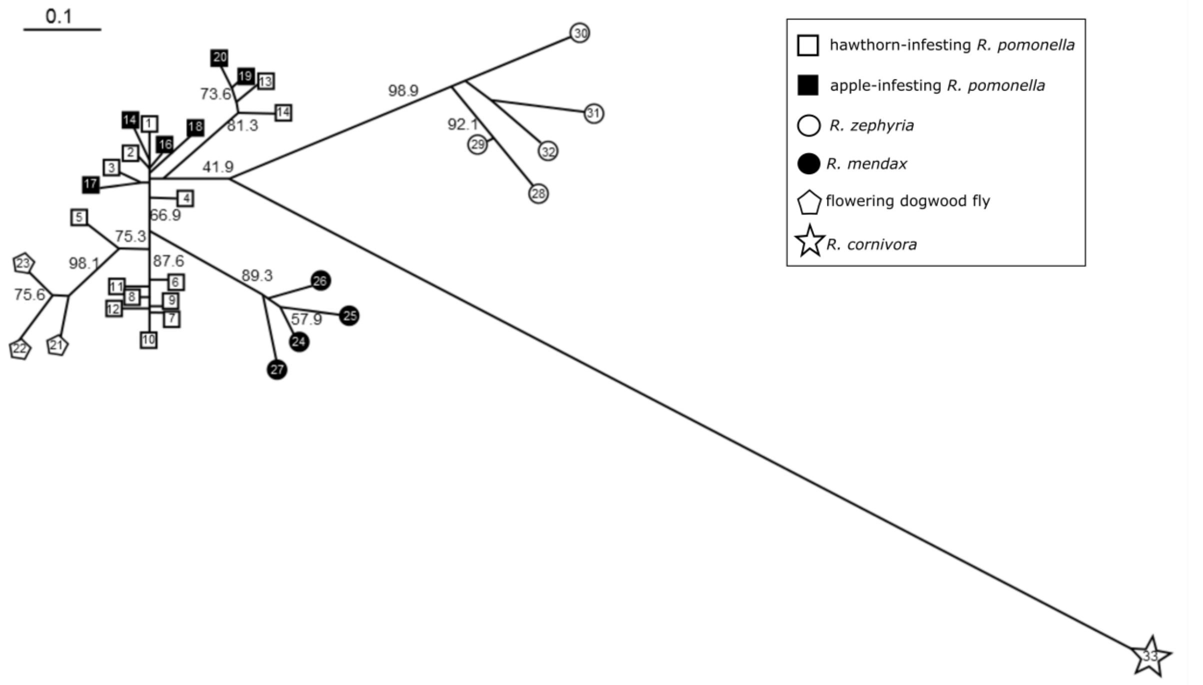

Figure S4. Neighbor-joining network based on Nei's D (1972) estimated from microsatellite allele frequencies for 19 loci. Open squares = hawthorn-infesting *R. pomonella*; closed squares = apple-infesting *R. pomonella*; open circles = *R. zephyria*; closed circles = *R. mendax*; pentagons = flowering dogwood fly, and star = *R. cornivora*. Bootstrap values come from 10,000 replicates across loci. Numbers within nodes refer to sites listed in Table 1 and Figure S2.

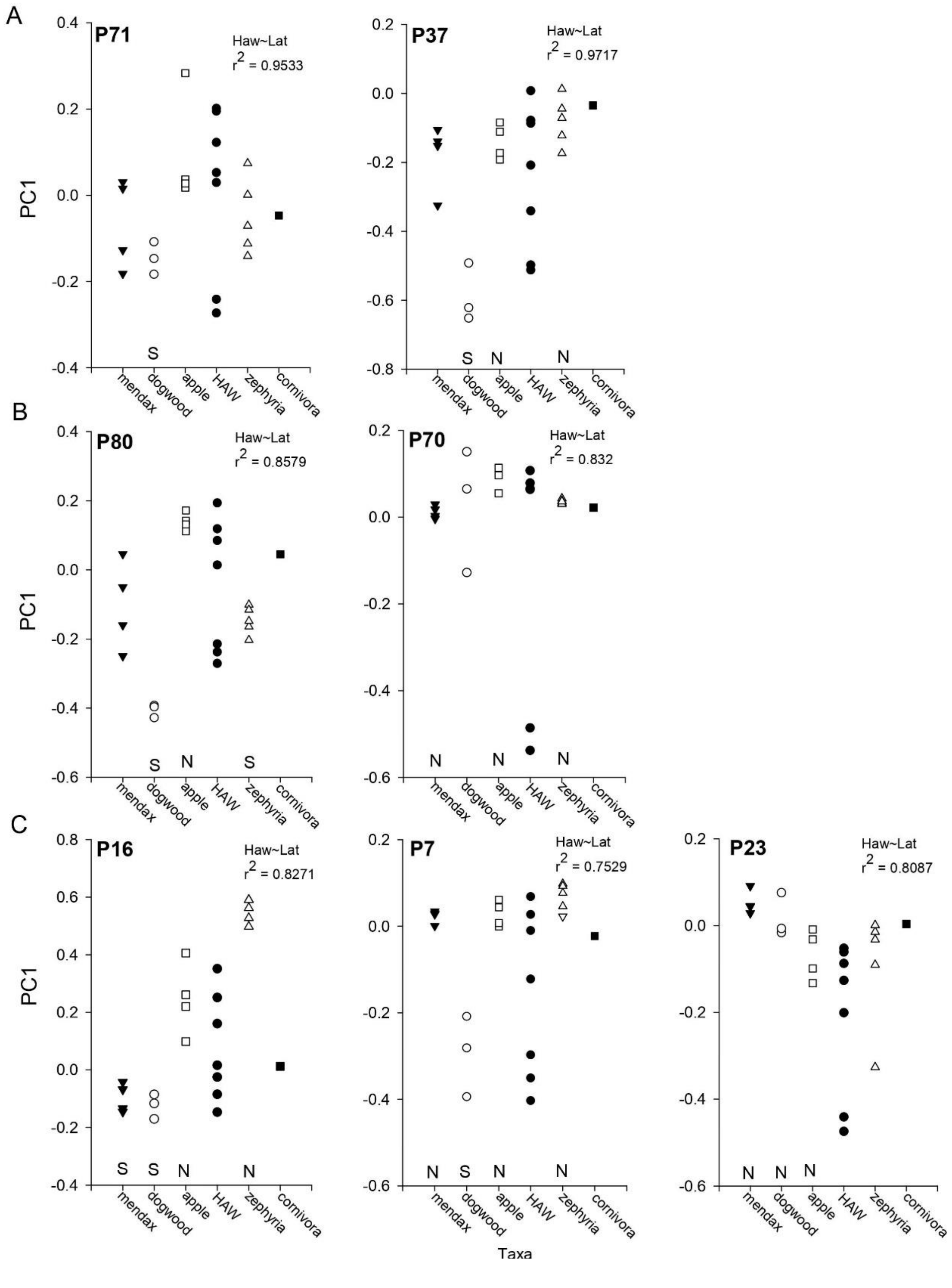

Figure S5. Principal component (PC1) scores of allele frequencies representing major clinal patterns within hawthorn-infesting *R. pomonella* populations (filled circles) for *R. mendax* (inverted triangles), the flowering dogwood fly (open circles), the apple race of *R. pomonella* (open squares), *R. zephyria* (open triangles), and *R. cornivora* (filled square) for seven major clinal loci on linkage groups 1 (A), 2 (B), and 3 (C). R^2 values reflect linear regression of hawthorn-race PC1 versus latitude. Capital letters along the x-axis designate taxa that were significantly northern-like (N) or southern-like (S) at that locus for across all populations, based lack of overlap and directionality of 95% confidence interval with estimated mean of PC1 between the taxa and the hawthorn race of *R. pomonella*.

Table S1. List of 19 microsatellite markers used in this study.

Locus	Chr	Genbank no.	Primer1 (5'-3')	Primer2 (5'-3')
P3	1	AY734887	TCCACTCAAATACGGCAACA	GCAGCCGATCTTTTCGTCTA
P4	1	AY734888	GCAAGCGAGTCGTAATCACA	CCCTCATCATTGTGGTCCTC
P7	3	AY734891	CATTGGCAACGCTAGTTCAA	GCGCTGAAACCATGAAAAAT
P9	5	AY734893	CGGCAGGTAAATGACCAAAA	GCAATGACCGTTGGCTATTA
P11	4	AY734895	ATGCAGCCATGACTGAGATG	TGGAAAGTAATTTCACAAAGGCTA
P16	3	AY734900	CGCTTTAGATTTTCGCTACACA	ACGCAGTGCCAAATCTTCTT
P18	5	AY734902	CCCAATGTCCCGTAAACTTC	TTCACTCAATGCCCATTTCA
P23	3	AY734907	AAACTGCCTTGCCTGTCATT	GCACTTTGTCGTTGATGCAC
P27	5	AY734911	TTCTCACATTTTCGCGTTTG	CTGGCCAATGCATAAATCCT
P29	4	AY734913	TCCATGTGTGCCAGAACATT	GACGTTATTTCGCTCGGTTG
P37	1	AY734921	CAACAGCGCGACTTAGTGAA	TGGCTTCCACCTTTGTTTTT
P46	2	AY734930	GCGCATTTCTCCATTCATTT	GCGGTAATTGTGCGTATGTG
P50	4	AY734934	GTGCAACCAGTGAGCAGTGT	TCTGACTGGCCCGTATTTGT
P60	4	AY734942	TACAACCTAGGCAGCCCAAC	GTCTGGTTTGGCGATCACTT
P66	3	AY734950	GCAAACCATTTTCCACGAAT	CGAAGCATGAATGCAACAAC
P70	2	AY734954	CAGCCTGCCAACACCATT	GCAACGCCTTCAAATTCATC
P71	1	AY734955	CGCAAGCACTTTTTGAACTG	CTGCTGAATTGGCAGCATAA
P73	2	AY734957	TTTTCTCGTCTACTCGTGTTAGTTAT	AAAATGCACTTTGTAAATAGTCACTCA
P80	3	AY734964	GGACAGTTGTGGTTGCTGAA	TCCTTTGCAATGTTATGGTAATTG

Table S2. Mean inbreeding coefficient (F_{IS}) across loci with 95% confidence intervals for each population, designations following Table 1.

Taxon	Population	mean F_{IS}	Lower 95% CI	Upper 95% CI
R. pomonella (Downy hawthorn)	1	0.138	-0.088	0.363
	2	0.119	-0.129	0.366
	3	0.132	-0.219	0.483
	4	0.109	-0.177	0.395
	5	0.038	-0.277	0.353
R. pomonella (green hawthorn)	6	0.075	-0.131	0.281
	7	0.017	-0.173	0.207
	8	0.06	-0.120	0.240
R. pomonella (blueberry hawthorn)	9	0.033	-0.290	0.356
	10	0.071	-0.126	0.268
R. pomonella (mayhaw)	11	0.077	-0.165	0.319
	12	0.106	-0.183	0.390
R. pomonella (black hawthorn)	13	0.081	-0.199	0.470
	14	0.138	-0.216	0.490
R. pomonella (Apple, eastern)	15	0.142	-0.117	0.401
	16	0.139	-0.132	0.411
	17	0.18	-0.149	0.509
	18	0.112	-0.108	0.332
R. pomonella (Apple, western)	19	0.149	-0.113	0.410
	20	0.153	-0.166	0.432
Flowering dogwood fly	21	0.091	-0.210	0.395
	22	0.023	-0.230	0.376
	23	0.089	-0.269	0.447
R. mendax	24	0.182	-0.306	0.670
	25	0.095	-0.232	0.422
	26	0.086	-0.130	0.302
	27	0.118	-0.239	0.475
R. zephyria	28	0.06	-0.410	0.533
	29	0.101	-0.361	0.584
	30	0.174	-0.236	0.583
	31	-0.073	-0.531	0.386
	32	0.073	-0.320	0.466
R. cornivora	33	0.169	-0.219	0.557

Table S3. Results of regional STRUCTURE runs establishing the presence of strong clustering patterns justifying subsequent migrant identification analyses, including mean estimated Ln Likelihood, standard deviation, and ΔK across five replicates, each run for a 750,000 iterations following a burn-in period of 500,000 iterations, of six values of K surrounding Likelihood plateaus identified from initial screen of K = 1 to K = total number of sites/host combinations of five replicates runs of 500,000 iterations following burn-in periods of 250,000 iterations for each of the three regions analyses.

	**Region**								
	**Northeast**			**South**			**Pacific Northwest**		
**K**	**mean LnP(K)**	**σ LnP(K)**	**ΔK**	**mean LnP(K)**	**σ LnP(K)**	**ΔK**	**mean LnP(K)**	**σ LnP(K)**	**ΔK**
1	-	-	-	-	-	-	-26732.5	0.403	-
2	-52375.77	320.95	-	-45201.35	0.71	-	-23426.9	0.360	8541.1
3	-51040.32	344.98	0.400	-44504.22	42.64	3.358	-23200.8	4.76	30.4
4	-49843.1	618.3	0.909	-43950.3	55.49	9.320	-23119.6	2.42	2.53
5	-49208.0	63.95	7.445	-43913.65	147.40	0.321	-23044.5	12.56	3.68
6	-49049.05	93.76	0.759	-43829.67	268.89	7.81	-22923.2	94.79	-
7	-48961.3	139.06	-	-45846.90	3080.11	-	-	-	-

Table S4. Count of putative parental migrants (P), F1 hybrids, and backcrosses (BX) among taxa of *R. pomonella* species complex members in the Northeastern United States. Counts represent individuals with the highest posterior probability of belonging to one of the migrant or hybrid classes as determined by STRUCTURE analysis. Receiving populations are listed in rows while source populations of migrants are listed in columns.

		**Source population**														
		R. pomonella			**Dogwood fly**			***R. mendax***			***R. zephyria***			***R. cornivora***		
**Rec. pop.**	***n***	**P**	**F1**	**BX**	**P**	**F1**	**BX**	**P**	**F1**	**BX**	**P**	**F1**	**BX**	**P**	**F1**	**BX**
R. pomonella	477				2	0	0	0	0	1	0	0	0	0	0	0
Dogwood fly	36	0	1	0				0	0	0	0	0	0	0	0	0
R. mendax	63	0	1	1	0	0	0				0	0	0	0	0	0
R. zephyria	87	0	0	1	0	0	0	0	0	1				0	0	0
R. cornivora	34	0	0	0	0	0	0	0	0	0	0	0	0			

Table S5. Count of putative parental migrants (P), F1 hybrids, and backcrosses (BX) among taxa of *R. pomonella* species complex members in the Southeastern United States. Counts represent individuals with the highest posterior probability of belonging to one of the migrant or hybrid classes as determined by STRUCTURE analysis. Receiving populations are listed in rows while source populations of migrants are listed in columns.

		**Source population**											
		R. pomonella			**Dogwood fly**			***R. mendax***			***R. cornivora***		
**Rec. pop.**	***n***	**P**	**F1**	**BX**	**P**	**F1**	**BX**	**P**	**F1**	**BX**	**P**	**F1**	**BX**
R. pomonella	466				0	0	0	0	1	0	0	0	0
Dogwood fly	77	1	0	0				0	0	0	0	0	0
R. mendax	57	0	0	0	0	0	0				0	0	0
R. zephyria	34	0	0	0	0	0	0	0	0	0			

Table S6. Count of putative parental migrants (P), F1 hybrids, and backcrosses (BX) among taxa of *R. pomonella* species complex members in the Pacific Northwest of the United States. Counts represent individuals with the highest posterior probability of belonging to one of the migrant or hybrid classes as determined by STRUCTURE analysis. Receiving populations are listed in rows while source populations of migrants are listed in columns.

		**Source population**					
		R. pomonella			***R. zephyria***		
**Rec. pop.**	***n***	**P**	**F1**	**BX**	**P**	**F1**	**BX**
R. pomonella	239				0	1	0
R. zephyria	145	1	0	0			

Table S7. Mean estimated Ln likelihood and standard deviation across ten replicates of STRUCTURE analysis of *R. pomonella* species complex taxa and paired sympatric *R. pomonella* populations a for K=1 and K=2, using a burn-in of 500,000 followed by 1,000,000 MCMC repetitions under a correlated allele frequency with admixture model. "Δ *Ln Lik*" reports the change in mean Ln likelihood between K=2 and K=1.

	**K=1**		**K=2**		
**Taxon**	***Ln Lik***	***σ***	***Ln Lik***	***σ***	**Δ *Ln Lik***
R. cornivora	-7103.5	0.91	-6127.7	0.64	-957.7
R. zephyria	-7217.2	1.16	-6225.1	0.48	-992.1
R. mendax	-8669.7	1.09	-8436.4	2.07	-233.29
Flowering dogwood fly	-6597.83	1.03	-6456.1	3.02	-141.74
Blueberry hawthorn fly	-10368.4	0.56	-10862.1	177.46	493.7
Apple fly	-7287.07	0.81	-7661.7	285.15	374.65
Mayhaw fly	-6779.9	0.90	-6838.7	47.27	58.81
